# Chromatin accessibility maps of chronic lymphocytic leukaemia identify subtype-specific epigenome signatures and transcription regulatory networks

**DOI:** 10.1038/ncomms11938

**Published:** 2016-06-27

**Authors:** André F. Rendeiro, Christian Schmidl, Jonathan C. Strefford, Renata Walewska, Zadie Davis, Matthias Farlik, David Oscier, Christoph Bock

**Affiliations:** 1 CeMM Research Center for Molecular Medicine of the Austrian Academy of Sciences, Lazarettgasse 14, 1090 Vienna, Austria; 2 Faculty of Medicine, Cancer Sciences, University of Southampton, Southampton SO17 1BJ, UK; 3 Department of Molecular Pathology, Royal Bournemouth Hospital, Bournemouth BH7 7DW, UK; 4 Department of Laboratory Medicine, Medical University of Vienna, 1090 Vienna, Austria; 5 Max Planck Institute for Informatics, 66123 Saarbrücken, Germany

## Abstract

Chronic lymphocytic leukaemia (CLL) is characterized by substantial clinical heterogeneity, despite relatively few genetic alterations. To provide a basis for studying epigenome deregulation in CLL, here we present genome-wide chromatin accessibility maps for 88 CLL samples from 55 patients measured by the ATAC-seq assay. We also performed ChIPmentation and RNA-seq profiling for ten representative samples. Based on the resulting data set, we devised and applied a bioinformatic method that links chromatin profiles to clinical annotations. Our analysis identified sample-specific variation on top of a shared core of CLL regulatory regions. *IGHV* mutation status—which distinguishes the two major subtypes of CLL—was accurately predicted by the chromatin profiles and gene regulatory networks inferred for *IGHV*-mutated versus *IGHV*-unmutated samples identified characteristic differences between these two disease subtypes. In summary, we discovered widespread heterogeneity in the chromatin landscape of CLL, established a community resource for studying epigenome deregulation in leukaemia and demonstrated the feasibility of large-scale chromatin accessibility mapping in cancer cohorts and clinical research.

## Abstract (web summary)

Chronic lymphocytic leukemia (CLL) is characterized by substantial clinical heterogeneity. Here, the authors report the genome-wide chromatin accessibility maps for 88 CLL samples from 55 patients using ATAC-seq, and 10 matched RNA-seq datasets, providing a resource for studying epigenome deregulation in CLL.

## Graphical abstract

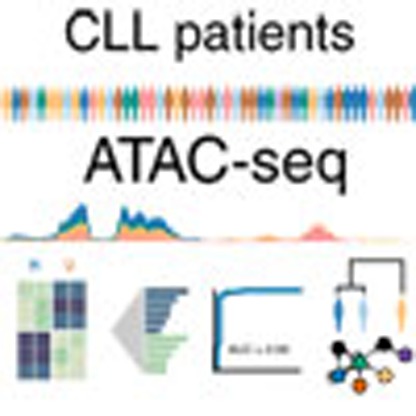

Chronic lymphocytic leukaemia (CLL) is the most common type of leukaemia in the Western world^1^. It is characterized by a remarkable clinical heterogeneity, with some patients pursuing an indolent course, whereas others progress rapidly and require early treatment. The diverse clinical course of CLL patients, in particular those that initially present with low disease burden, fuels interest in prognostic biomarkers and personalized therapies^2^. Current clinical biomarkers for CLL include mutational status of the *IGHV* genes^3^,^4^, *IGHV* gene family usage^5^, stereotyped B-cell receptors^6^,^7^, serum markers^8^,^9^, chromosomal aberrations^10^,^11^ and somatic mutations^12^,^13^,^14^. Most notably, *IGHV* mutation status distinguishes between a less aggressive form of CLL with mutated *IGHV* genes (mCLL) and a more aggressive form with unmutated *IGHV* genes (uCLL). Several surrogate biomarkers of *IGHV* mutation status have been described. For example, high levels of *ZAP70* expression appear to be associated with uCLL^15^. In addition to these focused biomarkers, transcriptome profiling has been used to define broader molecular signatures that may improve disease stratification independent of *IGHV* mutation status^16^.

Recent genome and exome sequencing projects have identified additional genes that are recurrently mutated in CLL^17^,^18^, some of which have prognostic significance. Nevertheless, CLL samples carry relatively few genetic aberrations compared with other adult cancers^19^, and some patients develop progressive disease despite being classified as ‘low risk’ based on genetic markers, suggesting that non-genetic factors are relevant for CLL aetiology and outcome. Several lines of evidence point to a role of epigenome deregulation in CLL pathogenesis: first, somatic mutations have been observed in non-coding regions of the genome, where they appear to induce deregulation of relevant cancer genes^18^. Second, chromatin remodelling proteins such as *ARID1A* and *CHD2* are recurrently mutated in CLL^17^,^18^, indicating causal links between chromatin deregulation and CLL. Third, aberrant DNA methylation was observed in all studied CLL patients^20^,^21^,^22^, correlated with *IGHV* mutation status and identified a new subtype (iCLL) that appears to be an intermediate between mCLL and uCLL^20^,^23^.

Although prior studies of epigenome deregulation in primary cancer samples have focused almost exclusively on DNA methylation^24^, recent technological advances now make it possible to map chromatin landscapes in large patient cohorts. Most notably, the assay for transposase-accessible chromatin using sequencing (ATAC-seq) facilitates open chromatin mapping in scarce clinical samples^25^ and ChIPmentation provides a streamlined, low-input workflow for genome-wide mapping of histone marks and transcription factors^26^. These two assays use a hyperactive variant of the prokaryotic Tn5 transposase, which integrates DNA sequencing adapters preferentially in genomic regions with accessible chromatin. ATAC-seq profiles are similar to those of DNase-seq, sharing the ability to detect footprints of transcription factor binding in the chromatin accessibility landscape^27^. ChIPmentation closely recapitulates the results obtained by more classical chromatin immunoprecipitation followed by sequencing protocols^26^. Both assays work well on scarce patient samples, and they enable fast sample processing on timescales that would be compatible with routine clinical diagnostics.

To establish the feasibility of large-scale chromatin analysis in primary cancer samples and to provide a basis for dissecting regulatory heterogeneity in CLL, we performed chromatin accessibility mapping using the ATAC-seq assay on a cohort of 88 primary CLL samples derived from 55 patients. Furthermore, for ten of these samples we established histone profiles using ChIPmentation for three histone marks (H3K4me1, H3K27ac and H3K27me3) and transcriptome profiles using RNA sequencing (RNA-seq). We also developed a bioinformatic method for linking these chromatin profiles to clinical annotations and molecular diagnostics data, and we performed an initial analysis of gene regulatory networks that underlie the major disease subtypes of CLL. In summary, this study provides a publicly available reference data set and a rich source of testable hypotheses for dissecting CLL biology and pathogenesis.

## Results

### Chromatin accessibility maps for 88 CLL samples

**Figure 1:**
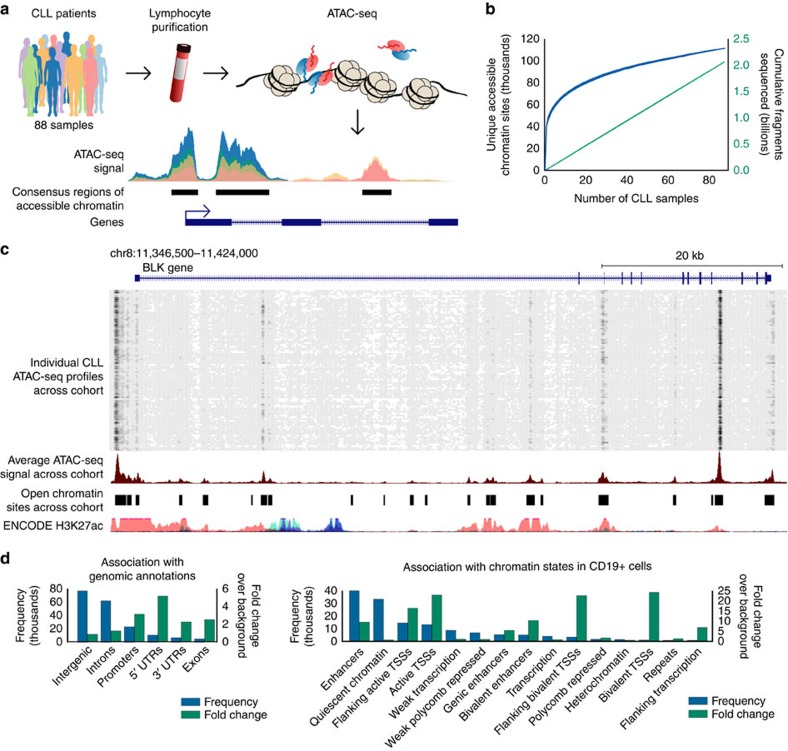
The chromatin accessibility landscape of CLL. (**a**) ATAC-seq profiling and analysis workflow for establishing patient-specific and cohort-level maps of chromatin accessibility in CLL. (**b**) Saturation analysis showing the number of unique chromatin-accessible regions detected across 88 samples and with a total sequencing depth of 2.2 billion ATAC-seq fragments. The narrow blue and green corridors indicate 95% confidence intervals for samples added in random order (1,000 iterations). (**c**) Genome browser plot showing ATAC-seq signal intensity for 88 individual CLL samples (top), average signal intensity across the cohort and cohort-level peak calls (centre) and reference data from the ENCODE project (bottom). Interactive genome browser tracks are available from the Supplementary Website: http://cll-chromatin.computational-epigenetics.org/. (**d**) Absolute (frequency) and relative (fold change) co-localization of unique chromatin-accessible regions in CLL with gene annotations (left) and chromatin state segmentations for CD19+ B cells from the Roadmap Epigenomics project (right).

To map the chromatin accessibility landscape of CLL (Fig. 1a), we performed ATAC-seq on 88 purified lymphocyte samples obtained from the peripheral blood of 55 CLL patients. These patients were managed at a single medical centre, and they collectively represent the spectrum of clinical phenotypes that are commonly observed in CLL (Supplementary Data 1). Their average age at sample collection was 73 years, and 8% of patients were sampled at relapse following initial or subsequent therapy. The majority of samples (58%) had been classified as *IGHV*-mutated as part of routine clinical diagnostics (Supplementary Fig. 1 and Supplementary Data 1).

All samples selected for ATAC-seq library preparation contained at least 80% leukaemic cells. The ATAC-seq libraries were sequenced with an average of 25.4 million fragments, resulting in a data set comprising a total of 2.2 billion sequenced fragments (Supplementary Data 2). Data quality was high in all cases, with mitochondrial read rates in the expected range for ATAC-seq (mean: 38.3%; s.d.: 9.3%) and the characteristic patterns of nucleosome phasing derived from paired-end data (Supplementary Fig. 2).

The individual samples were sequenced with sufficient depth to recover the majority of chromatin-accessible regions that are detectable in each sample (Supplementary Fig. 3). Moreover, by combining data across all 88 samples we approached cohort-level saturation in terms of unique chromatin-accessible regions (Fig. 1b), indicating that our cohort is sufficiently large to identify most regulatory regions commonly accessible in CLL samples.

As illustrated for the *BLK* gene locus (Fig. 1c), our ATAC-seq data set can be aggregated into a comprehensive map of chromatin accessibility in CLL. This map comprises 112,298 candidate regulatory regions, of which 11.6% are constitutively open across essentially all CLL samples, whereas 59.1% are open in a sizable proportion of samples (5–95% of samples) and 29.3% are unique to only one or very few samples (Supplementary Fig. 4a). All data are available for interactive browsing and download from the Supplementary Website (http://cll-chromatin.computational-epigenetics.org/).

Chromatin-accessible regions in CLL are widely distributed throughout the genome, with moderate enrichment at genes and promoters (Fig. 1d and Supplementary Fig. 4b). We also compared the CLL-accessible regions with epigenome segmentations for CD19+ B cells (Fig. 1e and Supplementary Fig. 4c), a related cell type for which comprehensive reference epigenome data are publicly available^28^. Strong enrichment was observed for regions that are classified as transcription start sites or as enhancer elements in the B cells, indicative of a globally similar chromatin accessibility landscape between B cells and CLL. Nevertheless, a sizable fraction of CLL-accessible regions carried quiescent or repressive chromatin in B cells, which is the expected pattern for regulatory elements that are subject to CLL-specific activation.

### Heterogeneity in the CLL chromatin accessibility landscape

**Figure 2:**
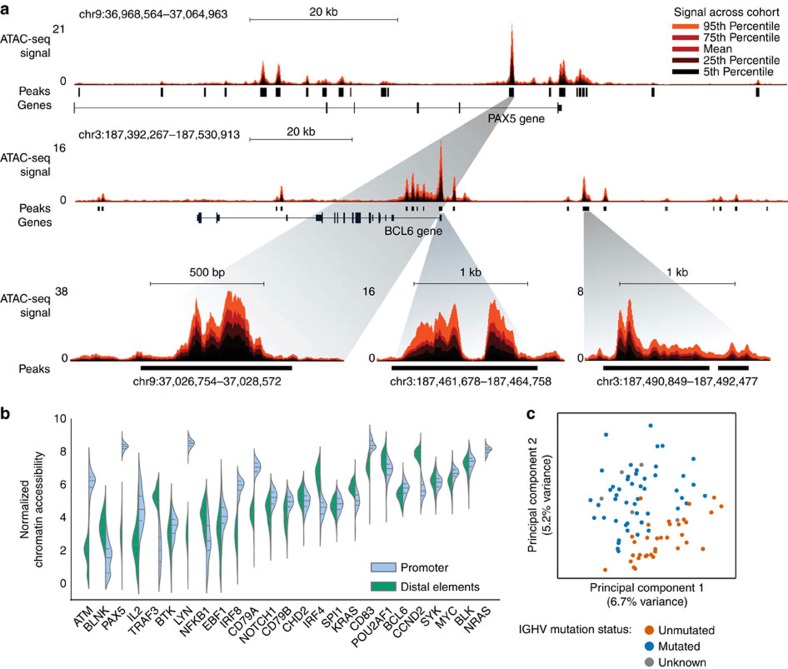
Heterogeneity in the chromatin accessibility landscape of CLL. (**a**) Genome browser plot showing ATAC-seq signal intensity across the CLL cohort in the vicinity of two genes with a known role in B-cell biology (*PAX5* and *BCL6*). This cohort-level track uses colour-coded percentiles to visualize the observed heterogeneity between samples. The bottom row zooms in on the chromatin accessibility landscape at three specific regulatory regions. (**b**) Violin plots showing the cohort-wide distribution of chromatin accessibility at promoters (chromatin-accessible regions located within 2,500 bp from the transcription start site) and putative enhancers of genes with a known role in B-cell biology and/or CLL pathogenesis. (**c**) Unsupervised principal component analysis based on the chromatin accessibility for all 88 samples at each of the 112,298 chromatin-accessible regions in the CLL cohort. Samples are colour coded according to their *IGHV* mutation status, using <98% germline homology as threshold for classifying a sample as mutated.

Although the number of constitutively accessible regions in our cohort was relatively low (11.6%, Supplementary Fig. 4a), we still observed high consistency between individual samples and, any two samples in our data set shared 70–98% of their chromatin-accessible regions (Supplementary Fig. 5a). Conversely, we also observed robust differences in the ATAC-seq signal intensity between samples. To facilitate gene-by-gene investigation of this heterogeneity, we established the ‘chromatin accessibility corridor’ as a means of aggregating the cohort-level variation into a single intuitive genome browser track (Fig. 2a and Supplementary Website). As illustrated by the *PAX5* and *BCL6* gene loci, even where the locations of chromatin accessible regions are shared across most samples, substantial differences in the ATAC-seq intensity levels were observed (Fig. 2a).

For a more systematic investigation of chromatin heterogeneity in CLL, we calculated the cohort-level variance for each of the 112,298 regions in the CLL consensus map and linked these regions to nearby genes that they may regulate (see Methods for details). Promoters of genes with a known role in B-cell biology and/or CLL pathogenesis showed significantly reduced variability (*P*<10^−5^, Kolmogorov–Smirnov test; Supplementary Fig. 5b), which was not due to differential representation of CpG islands among the promoters of the gene sets (*P*=0.49, Fisher’s exact test). For distal enhancer elements we did not observe any clear differences in heterogeneity between genes with and without a link to B cells and CLL (*P*=0.08, Kolmogorov–Smirnov test).

Beyond these global trends, the variance and distribution of chromatin accessibility across samples was highly gene specific (Fig. 2b and Supplementary Fig. 5c), as illustrated by CLL-linked genes including B-cell surface markers (*CD19*), B-cell receptor signalling components (*CD79A/B*, *LYN* and *BTK*), common oncogenes (*MYCN*, *KRAS* and *NRAS*) and genes that are recurrently mutated in CLL (*NOTCH1*, *SF3BP1*, *XPO1* and *CDKN1B*)^17^,^18^,^29^.

Unsupervised principal component analysis clearly identified *IGHV* mutation status as the major source of heterogeneity in chromatin accessibility among CLL samples (Fig. 2c and Supplementary Fig. 6). However, the first two principal components explained only 6.8 and 5.2% of the total variance in the chromatin accessibility data set, suggesting that many other factors contribute to the observed differences between samples.

The most direct way by which differences in chromatin accessibility may influence disease course would be through differential regulation of CLL-relevant genes. Therefore, to systematically assess the link between chromatin accessibility and gene expression in our cohort, we performed RNA-seq on ten CLL samples with matched ATAC-seq data. A weak positive correlation was observed between chromatin accessibility and gene expression (Pearson’s *r*=0.33; Supplementary Fig. 7a), which was highly dependent on the distance of the chromatin-accessible region to the nearest transcription start site (Supplementary Fig. 7b).

For chromatin-accessible regions in the vicinity of genes that RNA-seq identified as differentially expressed between *IGHV*-mutated (mCLL) and *IGHV*-unmutated (uCLL) samples (Supplementary Data 3), we observed significant differences in chromatin accessibility, which provided partial separation of the two disease subtypes (Supplementary Fig. 7c). A more pronounced separation was observed when we focused our analysis on those regions that had been identified as differentially methylated between mCLL and uCLL in a prior study of DNA methylation in CLL^20^ (Supplementary Fig. 7d).

Finally, we assessed whether patterns of differential variability between mCLL and uCLL (that is, higher levels of heterogeneity in one or the other subtype) may provide insights into the biology of these two disease subtypes. We identified 389 regions that showed a higher degree of variability among mCLL samples, whereas 581 regions were more variable among uCLL samples (Supplementary Fig. 8a)—consistent with prior results showing higher gene expression variability among uCLL samples^30^. These differentially variable regions were distributed across a broad range of ATAC-seq intensity values and were not a side effect of differences in average chromatin accessibility (Supplementary Fig. 8b). Genomic region enrichment analysis using the LOLA software^31^ found mCLL-variable regions enriched for B-cell-specific transcription factor binding (*ATF2*, *BATF*, *BCL6*, *NFKB* and *RUNX3*) and active histone marks (Supplementary Fig. 8c). In contrast, uCLL-variable regions were strongly associated with the cohesin complex, including binding sites for *CTCF*, *RAD21* and *SMC3*.

### Disease subtype-specific patterns of chromatin accessibility

**Figure 3:**
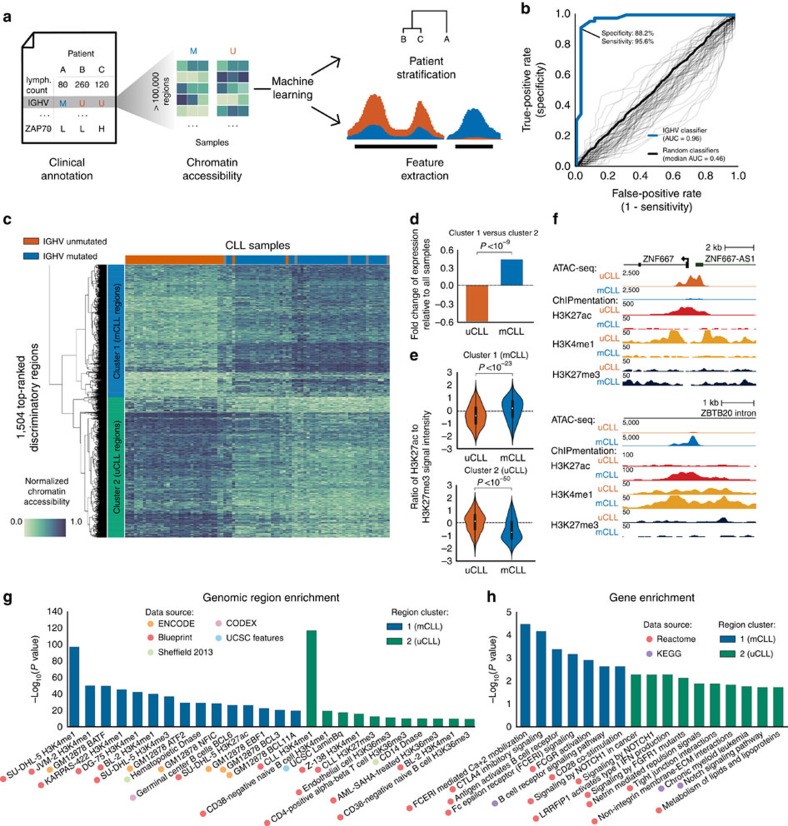
Disease subtype-specific patterns of chromatin accessibility. (**a**) Methodology for deriving disease subtype-specific patterns of chromatin accessibility: a machine learning algorithm is trained to distinguish between different sample sets (here *IGHV*-mutated versus *IGHV*-unmutated), the prediction performance is evaluated by cross-validation, and the most predictive features are obtained by feature extraction from the trained classifiers. (**b**) ROC curve summarizing the test set prediction performance (estimated by leave-one-out cross-validation) of a random forest classifier that uses the ATAC-seq data set to distinguish between *IGHV*-mutated and *IGHV*-unmutated samples. ‘AUC’ refers to the ROC area under curve as a measure of prediction performance, and sensitivity/specificity values are shown for the point on the ROC curve that is closest to the top left corner. The grey lines indicate the performance of 1,000 classifiers trained and evaluated in the same way but based on randomly shuffled class labels. (**c**) Clustered heatmap based on the most predictive regions extracted from the cross-validated classifiers. (**d**) Ratio of expression levels for genes linked to mCLL-accessible regions versus genes linked to uCLL-accessible regions. (**e**) Ratio between ChIPmentation signal for active chromatin (H3K27ac) and repressive chromatin (H3K27me3) at mCLL-linked and uCLL-linked regions. (**f**) Genome browser plots showing ATAC-seq and ChIPmentation profiles for gene loci with a known role in CLL (*ZNF667* and *ZBTB20*). (**g**) Most highly enriched region sets for mCLL (blue) and uCLL (green) associated regions. (**h**) Most highly enriched pathways among genes linked to mCLL (blue) and uCLL (green) regions.

To link the CLL chromatin accessibility landscape to clinical annotations and molecular diagnostics data (most notably to the *IGHV* mutation status that distinguishes between mCLL and uCLL), we devised a machine learning-based method that derives subtype-specific signatures directly from the data (Fig. 3a). Random forest classifiers were trained to predict whether a sample is *IGHV*-mutated or *IGHV*-unmutated, based on the chromatin accessibility values for all 112,298 regions in the CLL consensus map. We evaluated the performance of the resulting classifier by leave-one-out cross-validation and observed excellent prediction accuracy with a receiver operating characteristic (ROC) area under curve of 0.96 (Fig. 3b), which corresponds to a sensitivity of 95.6% at a specificity of 88.2%. To confirm that this cross-validated test set performance was not inflated by any form of overtraining, we repeated the same predictions one thousand times with randomly shuffled class labels. Much lower ROC area under curve values were observed in all cases, and their mean was very close to the theoretical expectation of 0.5 (Fig. 3b).

Next, we extracted the most predictive regions from the trained classifiers, giving rise to data-driven chromatin signatures that discriminate between mCLL and uCLL (Supplementary Data 4). Hierarchical clustering categorized these regions into 719 with increased chromatin accessibility in *IGHV*-mutated samples (‘mCLL regions’, cluster 1 in Fig. 3c) and 764 regions with increased chromatin accessibility in *IGHV*-unmutated samples (‘uCLL regions’, cluster 2 in Fig. 3c). More than half (51%) of these machine learning-based signature regions overlapped with statistically significant differential ATAC-seq peaks between *IGHV*-mutated and *IGHV*-unmutated samples (Supplementary Fig. 9a and Supplementary Data 4, see Methods for details). The remaining regions contributed to accurate prediction of CLL subtypes as part of a broader signature, even though they did not by themselves reach the stringent thresholds of the differential peak analysis (Supplementary Fig. 9b).

To test whether these subtype-specific chromatin signatures reflected more general differences in the gene regulatory landscape of CLL, we compared RNA-seq profiles and ChIPmentation maps for three histone marks (H3K4me1, H3K27ac and H3K27me3) between five *IGHV*-mutated and five *IGHV*-unmutated samples. We found that the genes in the vicinity of the signature regions were on average more highly expressed in the cell type showing higher chromatin accessibility (Fig. 3d and Supplementary Fig. 10), although only a small percentage of these genes were significantly differentially expressed between mCLL and uCLL samples based on our RNA-seq data (0.8% and 6.3%, respectively). Moreover, the ChIPmentation profiles were consistently associated with the differences in chromatin accessibility. Higher levels of the active H3K27ac mark as compared with repressive H3K27me3 were found in mCLL samples and mCLL-specific regions, and vice versa for uCLL (Fig. 3e). This observation is illustrated by the *ZNF667* promoter and an enhancer at the *ZBTB20* locus (Fig. 3f), two genes that have been identified as predictors of time to treatment and overall survival in CLL^32^,^33^.

Between individual samples we observed both qualitative (that is, the presence or absence of a peak) and quantitative (that is, different peak height) differences in chromatin accessibility, as illustrated by several genes with a known role in CLL (Supplementary Figs 11 and 12). For example, the expression ratio between *ADAM29* and *LPL* has been shown to have prognostic value in CLL^34^ and our data set identifies an mCLL-specific chromatin-accessible region within the *ADAM29* locus (Supplementary Fig. 11) as well as a uCLL-specific chromatin-accessible region overlapping with the *LPL* promoter (Supplementary Fig. 12), which may provide a regulatory basis for the previously described association. *CD83*, which has been associated with treatment-free survival^35^, is another example of a gene locus containing an mCLL-specific chromatin-accessible region (Supplementary Fig. 11). In contrast, uCLL-specific regions were identified in the gene loci encoding the CLL-linked transcription factor CREBBP^18^ and the surface protein CD38, which has been extensively validated as a prognostic factor in CLL^36^ (Supplementary Fig. 12).

To gain insight into the more general biological characteristics of the mCLL and uCLL signature regions, we performed genomic region set analysis using LOLA^31^ (Fig. 3g), and we observed that the mCLL regions were enriched for active promoter and enhancer regions (marked by H3K4me1 and H3K27ac) in lymphocyte-derived cell lines (SU-DHL-5, JVM-2, GM12878 and KARPAS-422), as well as binding sites of relevant transcription factors (BATF, BCL6 and BLC3). In contrast, the uCLL regions were enriched for H3K4me1-marked promoter/enhancer regions in CD38-negative naive B cells, reflecting the postulated naive B-cell origin of these CLL cells^37^. The uCLL regions were also enriched for transcribed regions (H3K36me3) in naive B cells and in B-cell-derived cell lines such as the BL-2 cell line, which has not undergone class-switch recombination.

We also performed motif enrichment analysis for the mCLL and uCLL signature region sets and, we observed significant enrichment relative to a random background model but no clear-cut differences when comparing the two region sets directly with each other (which is expected given the low statistical power of such an analysis). Nevertheless, when we linked chromatin-accessible regions to co-localized genes, we observed strong differences in the enrichment for cellular signalling pathways (Fig. 3h). The mCLL regions were associated with pathways having an established role in normal lymphocytes (CTLA4 inhibitory signaling, high-affinity IgE receptor signalling, Fc epsilon signalling and Fc gamma receptor signalling), whereas the uCLL regions were associated with cancer-associated pathways such as NOTCH signalling and fibroblast growth factor receptor signalling. All of these enrichment analyses were validated based on the statistically significant differential ATAC-seq peaks between *IGHV*-mutated and *IGHV*-unmutated samples, which gave rise to highly similar results (Supplementary Fig. 13).

**Figure 4:**
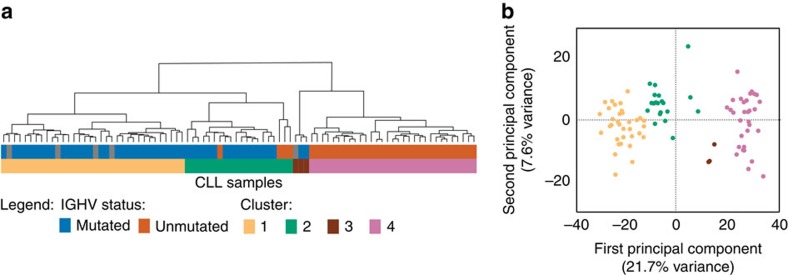
Patient stratification into CLL subtypes based on chromatin accessibility. (**a**) Hierarchical clustering of all CLL samples based the classifiers’ most predictive regions. Cluster 1 corresponds to mCLL, cluster 4 to uCLL, and the clusters 2 and 3 correspond to iCLL. Samples are coloured by *IGHV* mutation status (top) and cluster assignment (bottom). (**b**) Principal component analysis for the same data as in **a**, showing the first two principal components and their explained variance.

Finally, we investigated whether a third CLL subtype—termed *IGHV* intermediate (iCLL)—could be detected in our data set, as it was recently proposed based on DNA methylation data^20^,^23^. Clustering all samples based on the *IGHV* mutation signature regions, we indeed observed two intermediate clusters, the larger one comprising 20 samples from 14 patients (Fig. 4a, green) and the smaller one comprising 3 samples from 2 patients (Fig. 4a, brown). Most but not all of these iCLL samples were classified as *IGHV*-mutated based on the available molecular diagnostics data (Supplementary Fig. 14). Principal component analysis provided further evidence that the iCLL samples fall between mCLL and uCLL samples based on their ATAC-seq profiles (Fig. 4b). Their intermediate character was also supported by the RNA-seq and ChIPmentation data, where the iCLL group showed patterns that consistently ranged between those of the mCLL and uCLL groups (Supplementary Fig. 15).

### Gene regulatory networks in mCLL and uCLL disease subtypes

**Figure 5:**
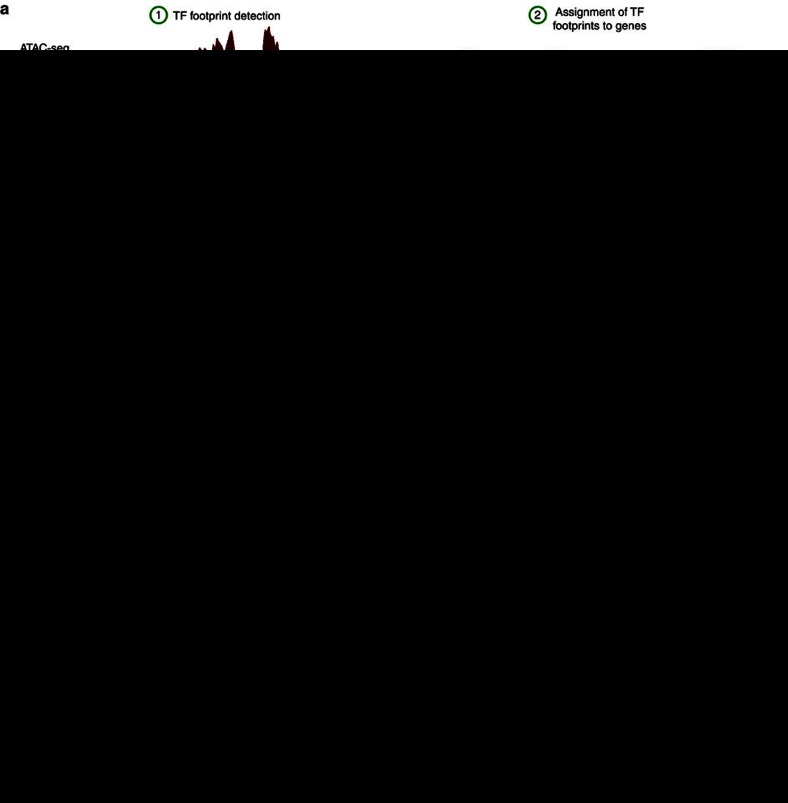
Gene regulatory networks underlying the mCLL and uCLL disease subtypes. (**a**) Methodology for deriving gene regulatory networks from ATAC-seq data using transcription factor (TF) footprinting, mapping of transcription factor-binding footprints to co-localized genes and regulatory network inference. (**b**) CLL gene regulatory network derived from the data of all 88 samples, showing the most differentially connected genes between uCLL and mCLL (the full network is shown in Supplementary Fig. 17). Node size reflects the total number of connections of each node, and colours denote the subtype-specific network in which the nodes are more highly connected (mCLL: blue; uCLL: orange). (**c**) Relative change in the number of connections between the mCLL and uCLL networks, showing all genes. (**d**) Relative change in the number of connections between the mCLL and uCLL networks, focusing on genes with a known role in B-cell biology and/or CLL pathogenesis.

In addition to providing chromatin accessibility maps, ATAC-seq can also detect transcription factor binding based on characteristic chromatin footprints^25^. Using this property of our data, we inferred chromatin-based gene regulatory networks for CLL and its two major disease subtypes (Fig. 5a). To that end, we pooled all ATAC-seq data across the analysed samples, identified footprints for 366 transcription factors with high-quality motifs in the JASPAR database^38^ and linked these regulatory elements to their putative target genes (see Methods for details). The quality of the observed footprints was comparable to those in publicly available DNase-seq data for CD19+ B cells (Supplementary Fig. 16), although we observed some deviations between the two assays that are likely due to the different sequence specificity of the Tn5 enzyme as opposed to the DNase I enzyme.

We first inferred a pan-CLL gene regulatory network using ATAC-seq data from all samples (Supplementary Fig. 17). The resulting network was dominated by highly connected transcription factors, including broadly activating factors (SP1/2/3), the insulator protein CTCF and regulators of biological processes such as cell proliferation (EGR), cell cycle (E2F) and B-cell maturation (SPI1 and PAX5). This pan-CLL network was structurally similar to a network for CD19+ B cells that we inferred from publicly available DNase-seq data using the same bioinformatic method (Supplementary Fig. 18), and in the absence of a large chromatin accessibility data set of B cells from healthy individuals it is not possible to conclusively identify the CLL-specific parts of our network.

Second, to investigate regulatory differences between CLL subtypes, we inferred gene regulatory networks separately for mCLL and uCLL samples (Supplementary Fig. 19) and identified the most differentially connected genes between the two (Fig. 5b). Genes that were more highly connected in the mCLL network coded for the transcription factors ZNF354C and ELF5, the metallopeptidase ADAM29 and the membrane protein CD22. In contrast, the BMP receptor CRIM1, the transcription factors MECOM and PAX9, the fibroblast growth factor signalling receptor FGFR1 and the membrane protein CD9 were more highly connected in the uCLL network (Fig. 5c). The more highly connected genes in either subtype also showed higher levels of H3K4me1 and H3K27ac in their regulatory elements in samples of the corresponding subtype (Supplementary Fig. 20a,b).

When we restricted our analysis to genes with a known role in B-cell biology and/or CLL pathogenesis (Fig. 5d), we observed a highly specific association of *CD22* (which codes for an inhibitory receptor involved in B-cell receptor signalling) with mCLL, whereas *CD38* and *ZAP70* were preferentially associated with uCLL. Focusing on *CD22* and *PAX9* as two high-ranking genes in our analysis, we plotted the sub-networks of their direct neighbours and observed characteristic differences between the gene regulatory networks for mCLL and uCLL (Supplementary Fig. 20c). Many of the subtype-specific genes identified by the regulatory network also showed locus-specific differences in their ChIPmentation profiles (Supplementary Fig. 20d). Altogether, our results confirm that ATAC-seq profiles are useful for identifying epigenome differences in clinical samples, and they illustrate how this data set can be used for deriving testable hypotheses about the regulatory basis of CLL.

## Discussion

By ATAC-seq profiling on a large set of primary CLL samples, we have established a detailed map of the chromatin accessibility landscape in CLL. The ATAC-seq data were complemented by RNA-seq profiles and ChIPmentation for three histone marks, performed in ten representative samples covering three disease subtypes (mCLL, uCLL and iCLL). To our knowledge, this data set is currently the largest catalogue of chromatin accessibility maps for any cancer type, demonstrating the feasibility of chromatin profiling in large cohorts of primary cancer samples and validating a broadly applicable bioinformatics workflow for analysing such data.

The large number of patient samples included in this study allowed us to dissect the role of epigenome variability as a potential contributor to cancer heterogeneity^39^. We found that variability between samples was common in our data set, both in the form of qualitative (that is, the presence or absence of a peak) and quantitative (that is, different peak height) differences between individual samples. In the absence of a reference data set with chromatin accessibility maps for normal B cells from a large number of healthy donors, it remains unclear whether or not the observed heterogeneity in CLL constitutes a major increase over the expected heterogeneity in a genetically diverse cohort. Nevertheless, significantly reduced heterogeneity at the promoters of genes involved in B-cell biology and/or CLL pathogenesis suggest a functional role of the observed inter-individual differences. Overall, our data support the existence of a core regulatory landscape shared by most or all CLL samples, which is complemented by sample-specific subsets of a substantially larger number of CLL-associated regulatory regions.

*IGHV* mutation status was the single biggest contributor to sample-specific differences in chromatin accessibility, although it explained only 5–10% of the observed variance in our data set. Based on the ATAC-seq profiles we were able to distinguish with excellent accuracy between *IGHV*-mutated mCLL and *IGHV*-unmutated uCLL. Our analysis also suggested the existence of one (or possibly two) intermediate type (iCLL), consistent with a recent report that used DNA methylation analysis of a large CLL cohort to identify novel CLL subtypes^20^. Chromatin accessibility and DNA methylation both appear to separate better between these disease subtypes than gene expression data, suggesting that the biological differences between the major subtypes of CLL are primarily encoded in the epigenome and possibly reflect patterns retained from a subtype-specific cell-of-origin.

Combining data across samples provided sufficient sequencing depth for footprinting analysis of transcription factor binding, allowing us to infer gene regulatory networks from the data and to compare them between mCLL and uCLL. Although genomic footprinting has its limitations^40^, the resulting network models give rise to predictions that can provide a starting point for further experimental dissection of the transcription regulatory landscape of CLL. For example, mCLL-associated regions were enriched for transcription factors that are active in mature B cells and involved in memory B-cell differentiation (BATF and BCL6), whereas the uCLL group was enriched for regulatory regions that are active in other haematopoietic cell types, indicative of a less differentiated cell state. Moreover, pathways that may boost proliferation, such as NOTCH signalling^41^ and interferon signalling^42^, were specifically observed in the more aggressive subtype (uCLL), whereas enrichment of inhibitory signalling by CTLA4 may contribute to the more indolent character of mCLL^43^. Beyond a small number of specific differences, the inferred gene regulatory networks were highly similar between mCLL and uCLL, consistent with the low number of differentially expressed genes that were previously observed between CLL subtypes^16^,^44^,^45^.

From a technological perspective, our study describes broadly applicable methods for dissecting chromatin profiles in large cohorts of primary patient samples. The differential chromatin analysis outlined in Fig. 3 starts from clinical and/or diagnostic data and uses supervised learning techniques to identify and cross-validate discriminatory chromatin signatures. We focused specifically on *IGHV* mutation status, but the method can be applied to any type of patient grouping, for example, based on disease progression or therapy response. Moreover, the described method for ATAC-seq-based inference of gene regulatory networks (Fig. 5) establishes a data-driven approach for dissecting regulatory cell states—including their differences between disease subtypes—that is highly complementary to previous work aimed at inferring regulatory networks from transcriptome data^46^,^47^,^48^. Finally, the ‘chromatin accessibility corridor’ (Fig. 2) adapts a related concept^49^ to provide intuitive browser-based visualization of chromatin data across large cohorts, while accounting for regulatory heterogeneity.

Relevant limitations of our study include the following: (i) lack of a clearly defined and experimentally accessible cell-of-origin for uCLL and mCLL, making it difficult to distinguish with certainty between chromatin patterns that are CLL specific and those that are derived from the disease's cell-of-origin; (ii) clonal heterogeneity of CLL within patients, which would be experimentally addressable only with single-cell sequencing technologies^50^,^51^ that are currently limited in their genome-wide coverage; (iii) lack of scalable methods for distinguishing between functional and non-functional transcription factor binding; and (iv) ambiguities in the assignment of transcription factor binding sites to the genes that they regulate. In the light of these limitations, the inferred gene regulatory networks constitute an initial model that will require future refinement as additional data and validations become available.

In summary, our study establishes a chromatin accessibility landscape of CLL, which identifies shared gene regulatory networks as well as widespread heterogeneity between individual patients and between disease subtypes. It also provides a resource that can act as a starting point for deeper dissection of chromatin regulation in CLL, identification of therapeutically relevant mechanisms and eventual translation of relevant discoveries into clinical practice. Given that the chromatin profiling assays used here (ATAC-seq and ChIPmentation) are sufficiently fast and straightforward for use in a clinical sequencing laboratory, chromatin deregulation is becoming increasingly tractable as a promising source of biomarkers for stratified cancer therapy.

## Methods

### Sample acquisition and clinical data

All patients were diagnosed and treated at the Royal Bournemouth Hospital (UK) according to the revised guidelines of the International Workshop Chronic Lymphocytic Leukemia/National Cancer Institute. Patients were selected to reflect the clinical and biological heterogeneity of the disease. Sequential samples were included for a total of 24 patients. All samples contained more than 80% leukaemic cells. Established chromosomal rearrangements were diagnosed by fluorescence *in situ* hybridization (Abbott Diagnostics; DakoCytomation) or multiple ligation-dependent probe amplification using the MLPA P037 CLL-1 probemix (MRC Holland SALSA) according to the manufacturers’ instructions. Chromosome analysis was performed and reported according to the International System for Human Cytogenetic Nomenclature. *IGHV* was sequenced as previously described^4^, and a threshold of >98% germline homology was taken to define the unmutated subset^4^. The study was approved by the ethics committees of the contributing institutions (Royal Bournemouth Hospital and Medical University of Vienna). Informed consent was obtained from all participants.

### ATAC sequencing

Accessible chromatin mapping was performed using the ATAC-seq method as previously described^25^, with minor adaptations. In each experiment, 10^5^ cells were washed once in 50 μl PBS, resuspended in 50 μl ATAC-seq lysis buffer (10 mM Tris-HCl pH 7.4, 10 mM NaCl, 3 mM MgCl_2_ and 0.1% IGEPAL CA-630) and centrifuged for 10 min at 4 °C. On centrifugation, the pellet was washed briefly in 50 μl MgCl_2_ buffer (10 mM Tris pH 8.0 and 5 mM MgCl_2_) before incubating in the transposase reaction mix (12.5 μl 2 × TD buffer, 2 μl transposase (Illumina) and 10.5 μl nuclease-free water) for 30 min at 37 °C. After DNA purification with the MinElute kit, 1 μl of the eluted DNA was used in a quantitative PCR (qPCR) reaction to estimate the optimum number of amplification cycles. Library amplification was followed by SPRI size selection to exclude fragments larger than 1,200 bp. DNA concentration was measured with a Qubit fluorometer (Life Technologies). Library amplification was performed using custom Nextera primers^25^. The libraries were sequenced by the Biomedical Sequencing Facility at CeMM using the Illumina HiSeq3000/4000 platform and the 25-bp paired-end configuration.

### RNA sequencing

Total RNA was isolated using the AllPrep DNA/RNA Mini Kit (Qiagen). RNA amount was measured using Qubit 2.0 Fluorometric Quantitation (Life Technologies), and the RNA integrity number was determined using Experion Automated Electrophoresis System (Bio-Rad). RNA-seq libraries were prepared using a Sciclone NGS Workstation (PerkinElmer) and a Zepyhr NGS Workstation (PerkinElmer) with the TruSeq Stranded mRNA LT sample preparation kit (Illumina). Library amount and quality were determined using Qubit 2.0 Fluorometric Quantitation (Life Technologies) and Experion Automated Electrophoresis System (Bio-Rad). The libraries were sequenced by the Biomedical Sequencing Facility at CeMM using the Illumina HiSeq 3000/4000 platform and the 50-bp single-read configuration.

### ChIPmentation

ChIPmentation was carried out as previously described^26^, with minor adaptions. Briefly, cells were washed once with PBS and fixed with 1% paraformaldehyde in up to 1 ml PBS for 10 min at room temperature. Glycine was added to stop the reaction. Cells were collected at 500 *g* for 10 min at 4 °C (subsequent work was performed on ice and used cool buffers and solutions unless otherwise specified) and washed twice with up to 0.5 ml ice-cold PBS supplemented with 1 μM phenylmethyl sulfonyl fluoride (PMSF). The pellet was lysed in sonication buffer (10 mM Tris-HCl pH 8.0, 1 mM EDTA pH 8.0, 0.25% SDS, 1 × protease inhibitors (Sigma) and 1 μM PMSF) and sonicated with a Covaris S220 sonicator for 20–30 min in a milliTUBE or microTUBE until the size of most fragments was in the range of 200–700 bp. Lysates were centrifuged at full speed for 5 min at 4 °C, and the supernatant containing the sonicated chromatin was transferred to a new tube. The lysate was then brought to RIPA buffer conditions (final concentration: 10 mM Tris-HCl pH 8.0, 1 mM EDTA pH 8.0, 140 mM NaCl, 1% Triton X-100, 0.1% SDS, 0.1% sodium deoxycholate, 1 × protease inhibitors (Sigma) and 1 μM PMSF) to a volume of 200 μl per immunoprecipitation. For each immunoprecipitation, 10 μl magnetic Protein A (Life Technologies) were washed twice and resuspended in PBS supplemented with 0.1% BSA. The antibody was added and bound to the beads by rotating 2 h at 4 °C. Used antibodies were H3K4me1 (0.5 μg per immunoprecipitation, Diagenode pAb-194-050), H3K27ac (1 μg per immunoprecipitation, Diagenode pAB-196-050) and H3K27me3 (1 μg per immunoprecipitation, Millipore 07-499). For control libraries, an immunoprecipitation with 2.5 μg of a nonspecific IgG rabbit antibody was used. Blocked antibody-conjugated beads were then placed on a magnet, supernatant was removed and the sonicated lysate was added to the beads followed by incubation for 3–4 h at 4 °C on a rotator. Beads were washed subsequently with RIPA (twice), RIPA-500 (10 mM Tris-HCl pH 8.0, 1 mM EDTA pH 8.0, 500 mM NaCl, 1% Triton X-100, 0.1% SDS and 0.1% DOC) (twice) and RIPA-LiCl (10 mM Tris-HCl pH 8.0, 1 mM EDTA pH 8.0, 250 mM LiCl, 1% Triton X-100, 0.5% DOC and 0.5% NP40) (twice).

Beads were washed once with cold Tris-Cl pH 8.0, to remove detergent, salts and EDTA. Beads were washed once more with cold Tris-Cl pH 8.0 but the reaction was not placed on a magnet to discard supernatant immediately. Instead, the whole reaction including beads was transferred to a new tube and then placed on a magnet to remove supernatant to decrease background. Beads were then carefully resuspended in 25 μl of the tagmentation reaction mix (10 mM Tris pH 8.0, 5 mM MgCl_2_, 10% v/v dimethylformamide) containing 1 μl Tagment DNA Enzyme from the Nextera DNA Sample Prep Kit (Illumina) and incubated at 37 °C for 1–3 min in a thermocycler. The beads were washed with RIPA (twice) and once with cold Tris-Cl pH 8. Beads were washed once more with cold Tris-Cl pH 8.0 but the reaction was not placed on a magnet to discard supernatant immediately. Instead, the whole reaction including beads was again transferred to a new tube and then placed on a magnet to remove supernatant. Beads were then incubated with 70 μl elution buffer (0.5% SDS, 300 mM NaCl, 5 mM EDTA and 10 mM Tris-HCl pH 8.0) containing 2 μl of Proteinase K (NEB) for 1 h at 55 °C and 8 h at 65 °C, to revert formaldehyde cross-linking, and supernatant was transferred to a new tube. Finally, DNA was purified with SPRI AMPure XP beads (sample-to-beads ratio 1:2) or Qiagen MinElute columns.

One microlitre of each library was amplified in a 10-μl qPCR reaction containing 0.15 μM primers, 1 × SYBR Green and 5 μl Kapa HiFi HotStart ReadyMix (Kapa Biosystems), to estimate the optimum number of enrichment cycles with the following programme: 72 °C for 5 min, 98 °C for 30 s, 24 cycles of 98 °C for 10 s, 63 °C for 30 s and 72 °C for 30 s, and a final elongation at 72 °C for 1 min. Kapa HiFi HotStart ReadyMix was incubated at 98 °C for 45 s before preparation of all PCR reactions (qPCR and final enrichment PCR), to activate the hot-start enzyme for successful nick translation at 72 °C in the first PCR step. Final enrichment of the libraries was performed in a 50-μl reaction using 0.75 μM primers and 25 μl Kapa HiFi HotStart ReadyMix. Libraries were amplified for *N*+1 cycles, where *N* is equal to the rounded-up Cq value determined in the qPCR reaction. Enriched libraries were purified using SPRI AMPure XP beads at a beads-to-sample ratio of 1:1, followed by a size selection using AMPure XP beads to recover libraries with a fragment length of 200–400 bp. Library preparation was performed using custom Nextera primers as described for ATAC-seq^25^. The libraries were sequenced by the Biomedical Sequencing Facility at CeMM using the Illumina HiSeq3000/4000 platform and the 25-bp paired-end configuration.

### Preprocessing of the ATAC-seq data

Reads were trimmed using Skewer^52^. Trimmed reads were aligned to the GRCh37/hg19 assembly of the human genome using Bowtie2 (ref. 53) with the ‘-very-sensitive’ parameter. Duplicate reads were removed using sambamba *markdup*^54^, and only properly paired reads with mapping quality >30 and alignment to the nuclear genome were kept. All downstream analyses were performed on the filtered reads. Genome browser tracks were created with the *genomeCoverageBed* command in BEDTools^55^ and normalized such that each value represents the read count per base pair per million mapped and filtered reads. Finally, the UCSC Genome Browser’s *bedGraphToBigWig* tool was used to produce a bigWig file. Combined tracks with percentile signal across the cohort were created by quantifying ATAC-seq read coverage at every reference genome position using BEDTools *coverage* and normalizing it between samples. Normalization was done by dividing each value by the total number of filtered reads and multiplying it with ten million, to obtain numbers that are comparable and easy to visualize. Next, the mean as well as the 5th, 25th, 75th and 95th percentiles of signal across the whole cohort were calculated with Numpy, converted into bedgraph files and subsequently to bigwig format using *bedGraphToBigWig*. Peak calling was performed with MACS2 (ref. 56) using the ‘-nomodel’ and ‘-extsize 147’ parameters, and peaks overlapping blacklisted features as defined by the ENCODE project^57^ were discarded.

### Preprocessing of the RNA-seq data

Reads were trimmed with Trimmomatic^58^ and aligned to the GRCh37/hg19 assembly of the human genome using Bowtie1 (ref. 59) with the following parameters: -q -p 6 -a -m 100—minins 0—maxins 5000—fr—sam—chunkmbs 200. Duplicate reads were removed with Picard’s *MarkDuplicates* utility with standard parameters before transcript quantification with BitSeq^60^ using the Markov chain Monte Carlo method and standard parameters. To obtain gene-level quantifications, we assigned the expression values of its highest expressed transcript to each gene. Differential gene-level expression between the three *IGHV* mutation status groups was performed using DESeq2 (ref. 61) from the raw count data with a significance threshold of 0.05. To produce genome browser tracks, we mapped the reads to the genomic sequence of the GRCh37/hg19 assembly of the human genome using Bowtie2 (ref. 53) with the ‘-very-sensitive’ parameter, removed duplicates using sambamba *markdup*^54^ and used the *genomeCoverageBed* command in BEDTools^55^ to produce a bedgraph file. This file was normalized such that each value represents the read count per base pair per million filtered reads, and the UCSC Genome Browser’s *bedGraphToBigWig* tool was used to convert it into a bigWig file.

### Preprocessing of the ChIPmentation data

Reads were trimmed using Skewer^52^. Trimmed reads were aligned to the GRCh37/hg19 assembly of the human genome using Bowtie2 (ref. 53) with the ‘-very-sensitive’ parameter. Duplicate reads were removed using sambamba *markdup*^54^, and only properly paired reads with mapping quality >30 and alignment to the nuclear genome were kept. All downstream analyses were performed on the filtered reads. Genome browser tracks were created with the *genomeCoverageBed* command in BEDTools^55^ and normalized such that each value represents the read count per base pair per million filtered reads. Finally, the UCSC Genome Browser’s *bedGraphToBigWig* tool was used to produce a bigWig file.

### Bioinformatic analysis of chromatin accessibility

The CLL consensus map was created by merging the ATAC-seq peaks from all samples using the BEDTools^55^ *merge* command. To produce Fig. 1b, we counted the number of unique chromatin-accessible regions after merging peaks for each sample in an iterative manner, randomizing the sample order 1,000 times and computing 95% confidence intervals across all iterations. The chromatin accessibility of each region in each sample was quantified using Pysam, counting the number of reads from the filtered BAM file that overlapped each region. To normalize read counts across samples, we performed quantile normalization using the *normalize.quantiles* function from the preprocessCore package in R. For each genomic region we calculated the support as the percentage of samples with a called peak in the region, and we calculated four measures of ATAC-seq signal variation across the cohort: mean signal, s.d., variance-to-mean ratio and the squared coefficient of variation (the square of the s.d. over the mean). In addition, we used BEDTools *intersect* to annotate each region with the identity of and distance to the nearest transcription start site and the overlap with Ensembl gene annotations (promoters were defined as the 2,500-bp region upstream of the transcription start site). Annotation with chromatin states was based on the 15-state genome segmentation for CD19+ B cells from the Roadmap Epigenomics Project^62^ (identifier: E032).

To summarize the chromatin accessibility signals into one value per gene (Fig. 2b and Supplementary Fig. 5), we used the accessibility values of the closest region (but no further than 1,000 bp from the transcription start site) to represent the promoter and the mean values of all distal regions (located more than 2,500 bp from the transcription start site) of each gene to represent distal regulatory elements. To test for overrepresentation of CpG islands in the promoters of genes with a known role in B-cell biology and/or CLL pathogenesis, we downloaded the position of CpG islands in the GRCh37/hg19 assembly from the UCSC Genome Browser^63^, counted the number of promoters (as defined above) that overlapped by at least 1 bp with CpG islands in the gene set of interest and in all other genes with accessible elements in CLL, and used Fisher’s exact test to assess the significance of the association. Unsupervised principal component analysis was performed with the scikit-learn^64^ library (*sklearn.decomposition.PCA)* applied to the chromatin accessibility values of all chromatin-accessible regions across the CLL cohort.

To investigate variability within the mCLL and uCLL sample groups, we divided the samples in two groups based on their *IGHV* mutation status (samples below a 98% homology threshold were considered mutated, and samples with missing values for the *IGHV* mutation status were excluded from the analysis) and we used the F test from the *var.test* function in R on the chromatin accessibility values of all CLL cohort regions. Significantly variable regions were defined as having a Bonferroni-corrected *P*-value below 0.05 and mean accessibility above 1. Region set-enrichment analysis was performed on the significantly variable regions of each group using LOLA^31^ with its core databases: transcription factor binding sites from ENCODE^57^, tissue clustered DNase hypersensitive sites^65^, the CODEX database^66^, UCSC Genome Browser annotation tracks^63^, the Cistrome database^67^ and data from the BLUEPRINT project^68^. Motif enrichment analysis was performed with the AME tool from the MEME suite^69^ using 250 bp sequences centred on the chromatin-accessible regions and randomly generated sequences of the same length and set size from a distribution of zeroth- and first-order Markov order (single nucleotides and dinucleotide) frequencies as background.

### Machine learning analysis of disease subtypes

Random forest classifiers from the scikit-learn^64^ Python library (*sklearn.ensemble.RandomForestClassifier*) were trained with the samples’ *IGHV* mutation status as class label and the chromatin accessibility values for each sample at each of the 112,298 consensus regions as input features (prediction attributes). All samples with known *IGHV* mutation status were used for class prediction, the performance was evaluated by leave-one-out cross-validation, and the results were plotted as ROC curves using scikit-learn. Given that several patients contributed more than one sample to the cohort, in each iteration of the cross-validation we removed any samples from the training set that belonged to the same patient as the sample in the test set, to eliminate a potential risk of overtraining. Furthermore, we repeated the cross-validation 1,000 times based on randomly shuffled class labels to confirm that no overtraining occurred in our analysis. The most predictive regions for *IGHV* mutation status were selected by averaging the feature importance of the random forest classifiers over all iterations of the cross-validation and selecting those features with Gini importance higher than 10^−4^. Region set enrichment was performed using LOLA^31^ as described above. Pathway enrichment analysis was performed using seq2pathway^70^. The sample clustering in Fig. 4a was based on the pairwise correlation of ATAC-seq signal in the predictive regions between samples, and the dendrogram was plotted using Scipy’s hierarchical clustering function. With the same values of chromatin accessibility from above, we performed principal component analysis on the CLL samples using R’s implementation in the *prcomp* function. To provide further validation of the machine learning analysis, we also identified differential ATAC-seq peaks between *IGHV*-mutated and *IGHV*-unmutated samples using the DESeq2 R package^61^. This statistical analysis was based on read counts for all CLL-accessible regions in each patient, testing for differential chromatin accessibility using a model based on the negative binomial distribution. Regions with Benjamini–Hochberg adjusted *P*-values below 0.01 and an absolute log2 fold change above 1 were used for comparison with those signature regions identified by the machine-learning analysis.

### Gene regulatory network inference

Transcription factor binding maps as the basis for inferring gene regulatory networks were derived by footprinting analysis using the PIQ software^71^ and a set of 366 human transcription factor motifs from the JASPAR database^38^. We retained only those transcription factors with at least 500 high-purity (>0.7) binding sites overlapping with an ATAC-seq peak, as previously described^72^. Binding sites located in the gene body or in the 2,500-bp region upstream of its transcription start site were assigned to the overlapping gene(s), and intergenic binding sites were assigned to the gene whose transcription start site was closest to the peak. This assignment was based on the Ensembl gene annotation version 75, and we treated non-protein-coding genes in the same way as protein-coding genes. To infer gene regulatory networks, an interaction score was calculated in a similar way as previously described^72^: the interaction score between a transcription factor *t* and a gene *g* (*S*_t,g_) was defined as the sum over all *n* transcription factor binding sites of *t* that can be assigned to *g*:


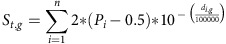


In this formula *P*_*i*_ is the PIQ purity score, and *d*_*i,g*_ is the distance of a particular transcription factor binding site *i* to gene *g*. This score establishes a unidirectional (transcription factors to genes) and weighted (based on the interaction score) relationship, providing the edges of the gene regulatory network. We inferred gene regulatory networks for all samples combined and also separately for the two disease subtypes (mCLL and uCLL) based on *IGHV* mutation status. We considered only transcription-factor-to-gene interactions with scores above 1, and in Fig. 5b and Supplementary Figs 17 and 19 we plotted only nodes with more than 200 connections. For the CD19+ B-cell gene regulatory network we used DNase-seq data from the Roadmap Epigenomics Project^62^ (identifier: E032). Both the processing of the raw data and the network inference were performed in the same manner as for ATAC-seq. The comparison of composition and structural characteristics of the gene regulatory networks inferred from ATAC-seq data for the CLL cohort and from DNase-seq data for CD19+ B cells was done using functions from the *networkx*^73^ library in Python. The inferred networks were visualized using the Gephi software, applying the Force Atlas 2 graph layout with LinLog and hub dissuasion. To compare the inferred mCLL and uCLL networks, we divided the degree of each node by the total number of edges in each network, which compensates for differences in the absolute number of detected interactions, and quantified differences by subtracting and log2-transforming this value between networks for each node.

### Data availability

All data are available as genome browser tracks for interactive browsing and download from the Supplementary Website (http://cll-chromatin.computational-epigenetics.org/). The processed data are also openly available from NCBI GEO under the accession number GSE81274, whereas the raw sequencing data are available from EBI EGA under the accession number EGAS00001001821, under a controlled access regimen to protect the privacy of the patients who have donated the samples.

## Additional information

**Accession codes:** The processed high-throughput sequencing data are openly available from NCBI GEO under the accession number GSE81274, whereas the raw sequencing data are available from EBI EGA under the accession number EGAS00001001821, under a controlled access regimen to protect the privacy of the patients who have donated the samples.

**How to cite this article:** Rendeiro, A. F. *et al.* Chromatin accessibility maps of chronic lymphocytic leukaemia identify subtype-specific epigenome signatures and transcription regulatory networks. *Nat. Commun.* 7:11938 doi: 10.1038/ncomms11938 (2016).

## Supplementary Material

Supplementary Information Supplementary Figures 1-20

Supplementary Data 1 Clinical annotations of the CLL patient cohort. Clinical annotations for the patient samples that were analyzed in this study. All patients were diagnosed and treated at the Royal Bournemouth Hospital (UK).

Supplementary Data 2 Summary statistics of the sequencing experiments. Sequencing statistics for 88 samples with ATAC-seq, 10 samples with ChIPmentation for three histone marks (H3K4me1, H3K27ac, H3K27me3) and one control (IgG), and 10 samples with RNA-seq.

Supplementary Data 3 Differentially expressed genes between CLL subtypes. List of differentially expressed genes between three disease subtypes (mCLL, iCLL, uCLL), based on RNA-seq data for representative samples in each subtype.

Supplementary Data 4 Chromatin-accessible regions associated with IGHV mutation status. List of regions with differential chromatin accessibility between IGHV-mutated and IGHV-unmutated CLL samples, based on the machine learning analysis or alternatively based on differential peak analysis using DESeq2.
