# Supplementary material for: Chromatin accessibility maps of chronic lymphocytic leukaemia identify subtype-specific epigenome signatures and transcription regulatory networks
Source: Nat Commun. 2016 Jun 27;7:11938. doi: 10.1038/ncomms11938 (PMC5494194; doi:10.1038/ncomms11938)
Supplement: Supplementary Information — Supplementary Figures 1-20 [file ncomms11938-s1.pdf]

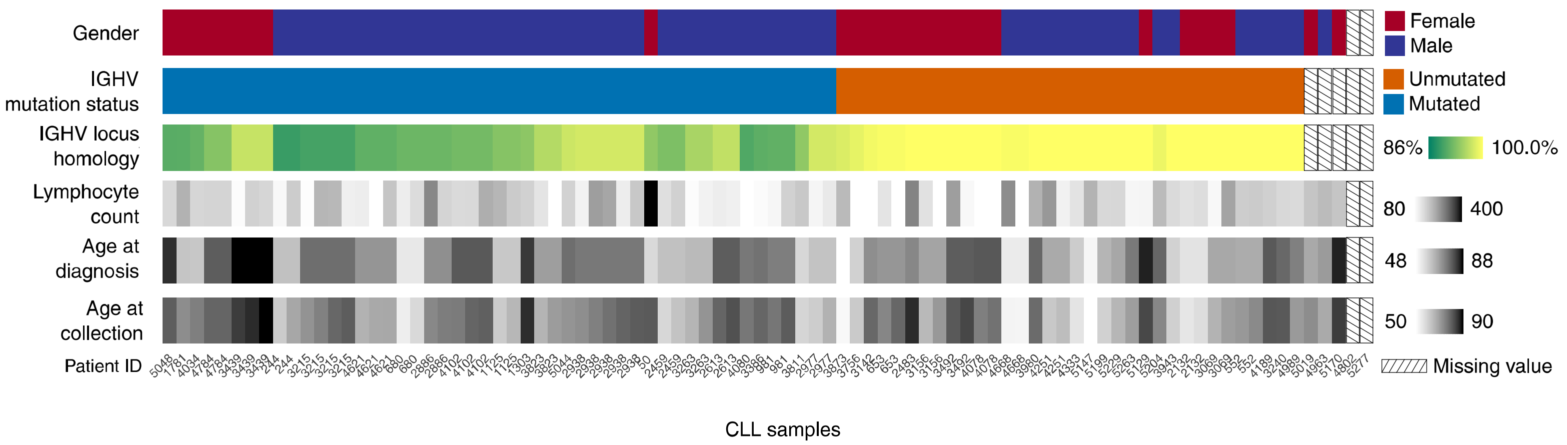

Supplementary Figure 1

The cohort reflects the spectrum of CLL phenotypes commonly encountered in clinical care.

Visualization of clinical annotations for the patient samples included in this study.

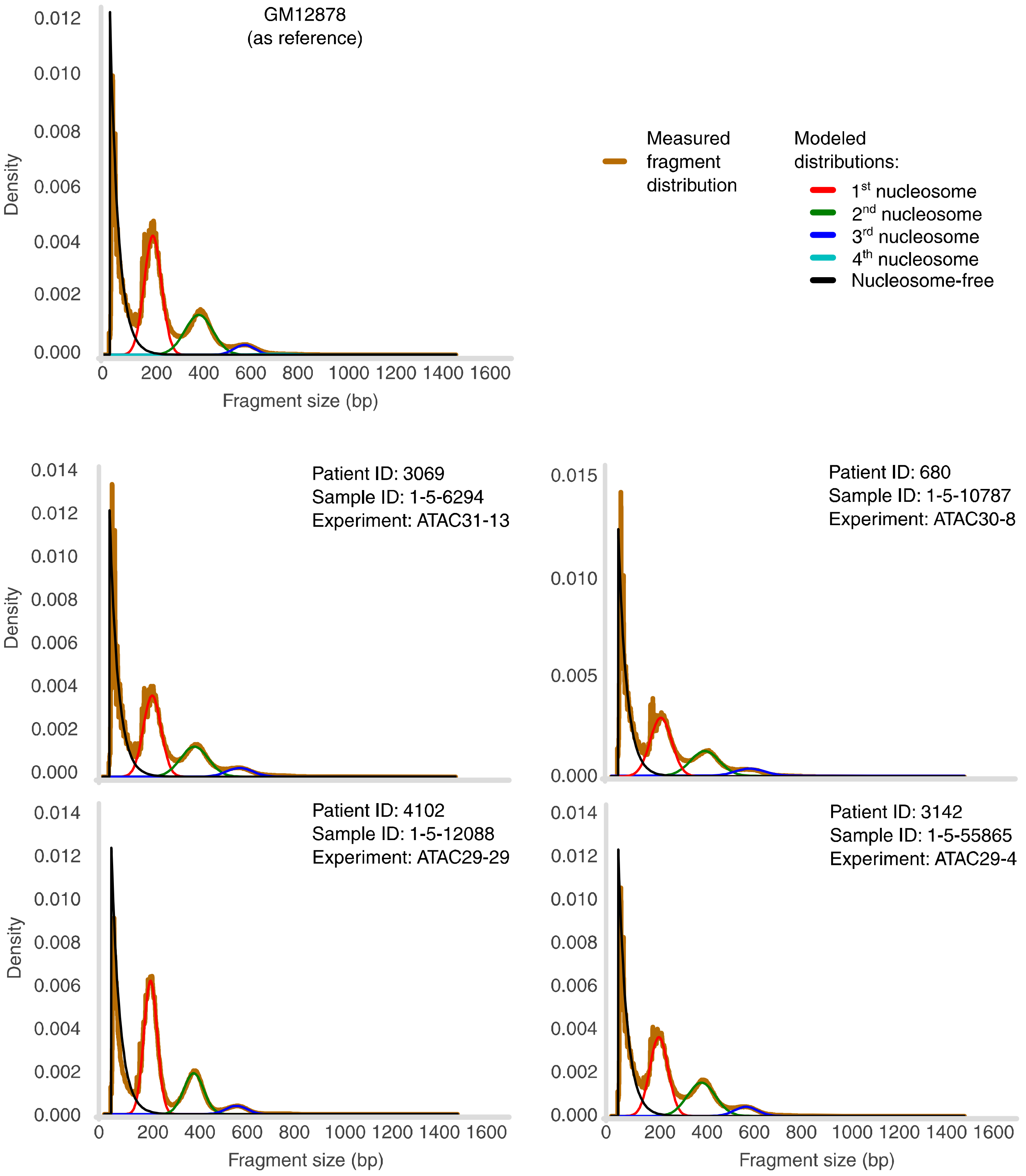

Supplementary Figure 2

Observed ATAC-seq fragment length distributions indicate high data quality.

Distribution of ATAC-seq fragment lengths for published GM12878 data (Buenrostro et al. 2013 Nature Methods) and for four randomly selected CLL samples from this study. Fragment lengths were inferred based on paired-end sequencing data. The characteristic patterns of nucleosome-associated fragment length are observed in all samples.

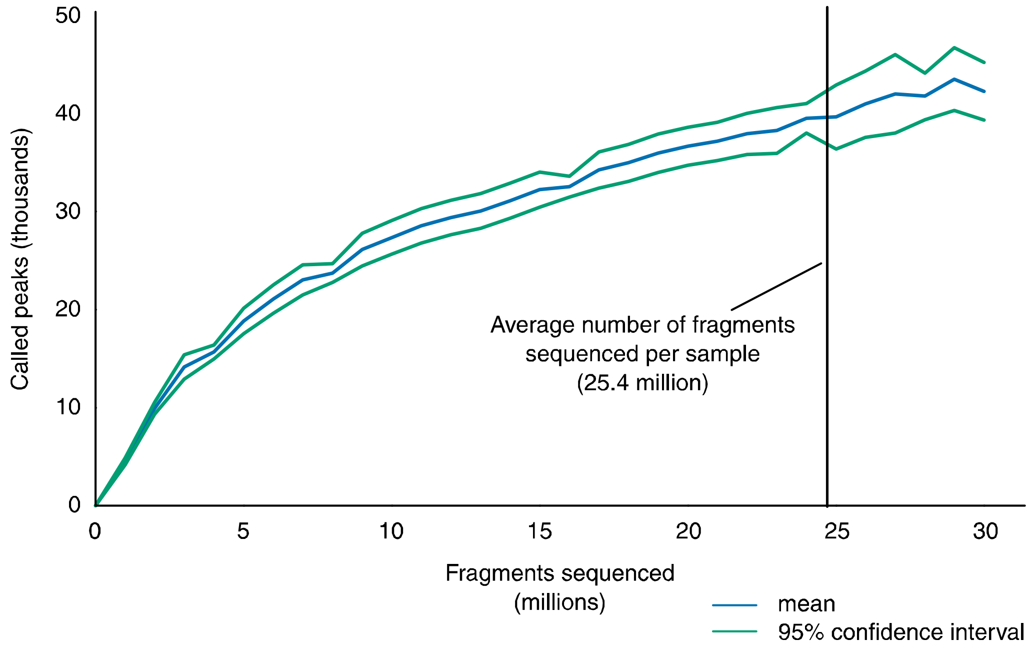

Supplementary Figure 3

The chosen sequencing depth recovers the majority of ATAC peaks per sample.

Relationship of the number of sequenced reads (x-axis) and the number of detected chromatin-accessible regions (y-axis), showing the average pattern across all 88 samples (blue line). The corridor indicated in green corresponds to a 95% confidence interval for random subsampling across samples.

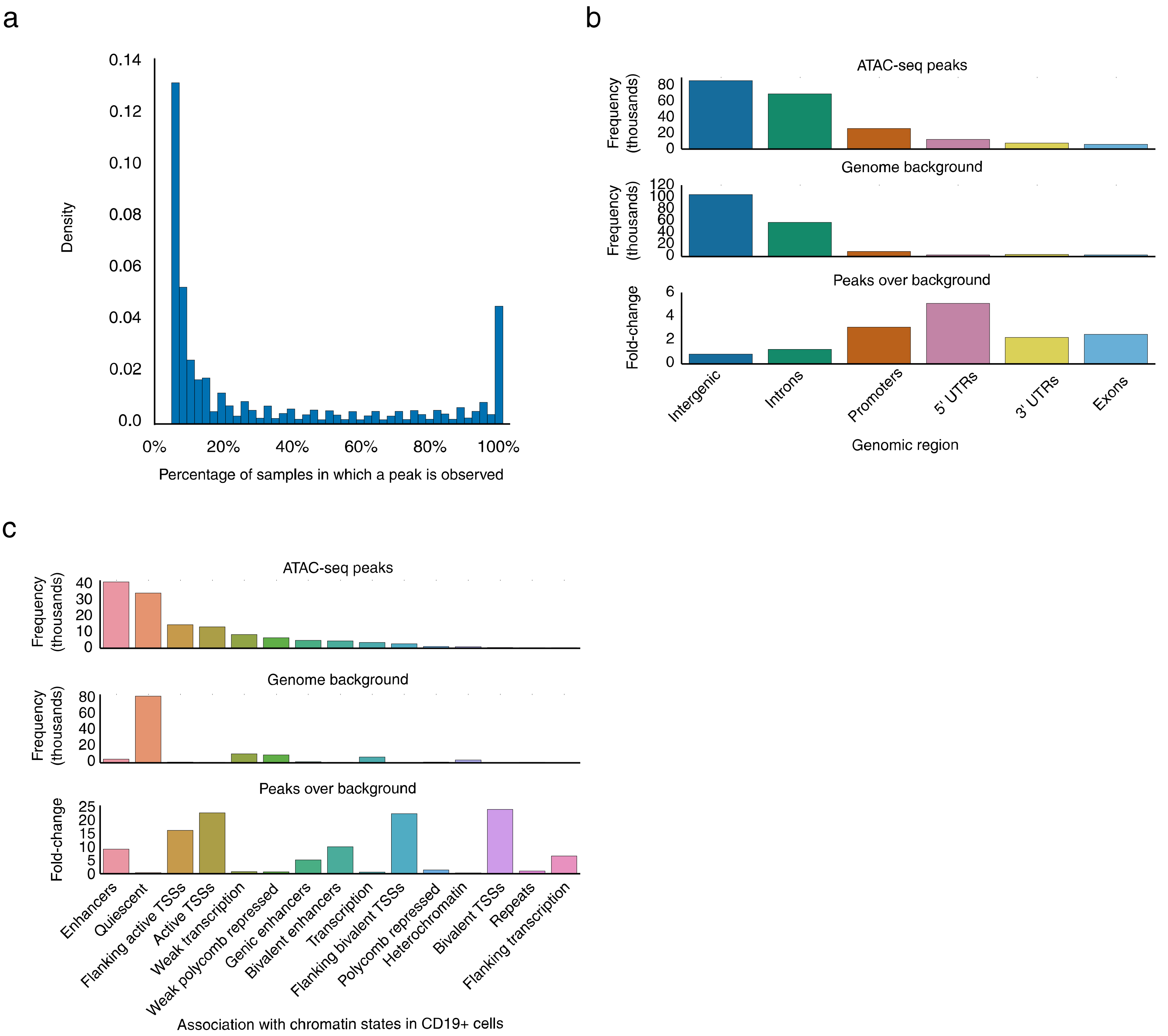

Supplementary Figure 4

Chromatin-accessible regions in CLL are enriched for promoters and enhancers.

a) Histogram showing the number of samples in which a given chromatin-accessible region from the CLL consensus map was detected as a significant ATAC-seq peak. b) Frequency of overlap and enrichment of Ensembl gene annotation for regions in the CLL consensus map, compared to region sets of identical size and lengths that were randomized 1,000 times across the genome. c) Frequency of overlap and enrichment of chromatin state segmentations for CD19+ B cells (data from the Roadmap Epigenomics project), compared to region sets of identical size and lengths that were randomized 1,000 times across the genome.

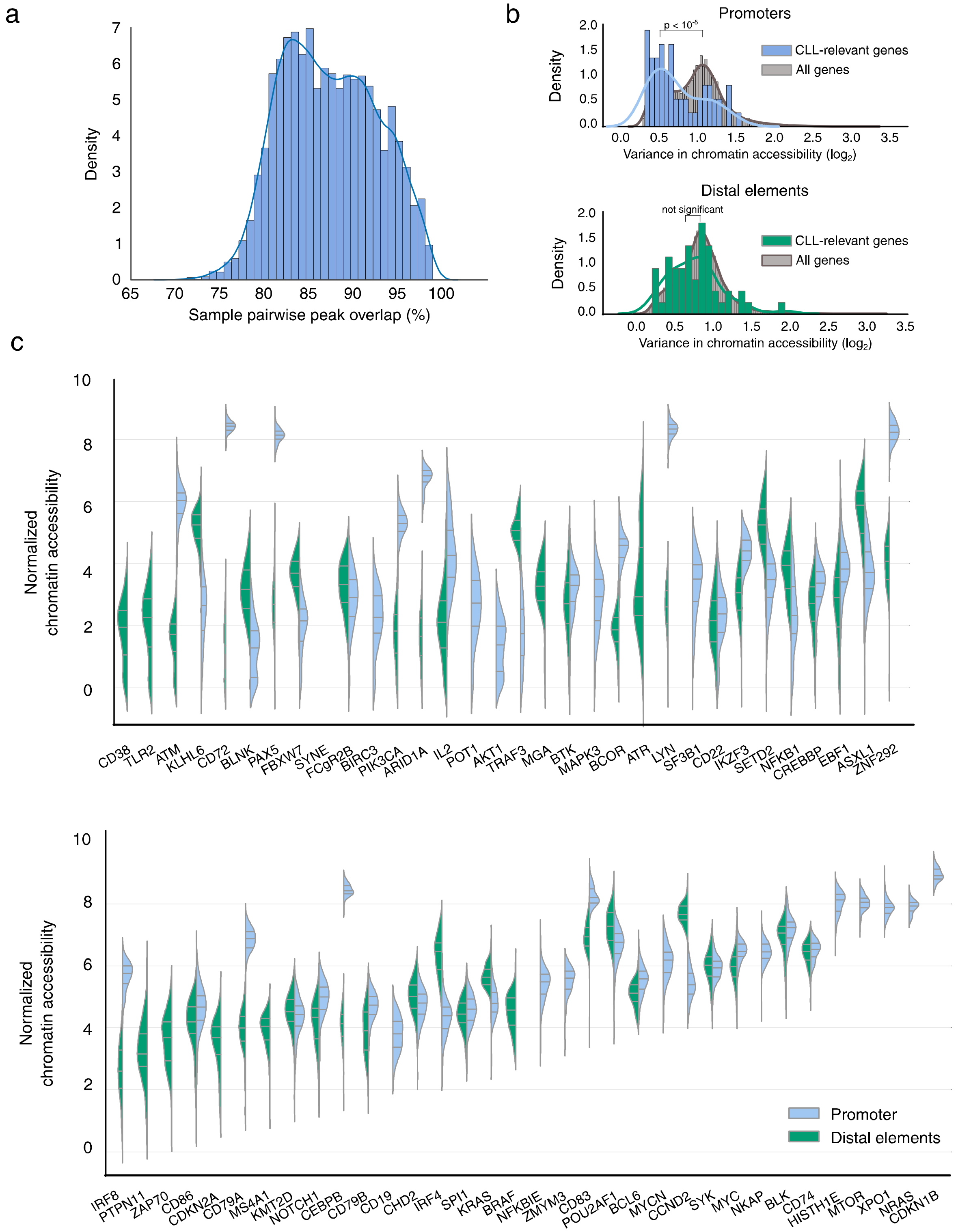

Supplementary Figure 5

Heterogeneity in chromatin accessibility affects genes related to B cells and CLL.

a) Histogram showing the percentage of chromatin-accessible regions that are shared between any two CLL samples. b) Distribution of variance in chromatin accessibility for promoter regions and putative distal regulatory regions across all genes (grey) and for a set of genes with a known role in B cell biology and/or CLL pathogenesis (blue/green). Chromatin accessibility scores were averaged across all regulatory regions assigned to a given gene. c) Violin plots of normalized chromatin accessibility values for gene promoters (regions located within 2,500 basepairs of the transcription start site) and distal regulatory elements (regions located at least 2,500 basepairs away from the nearest transcription start site) for the same genes as in panel b.

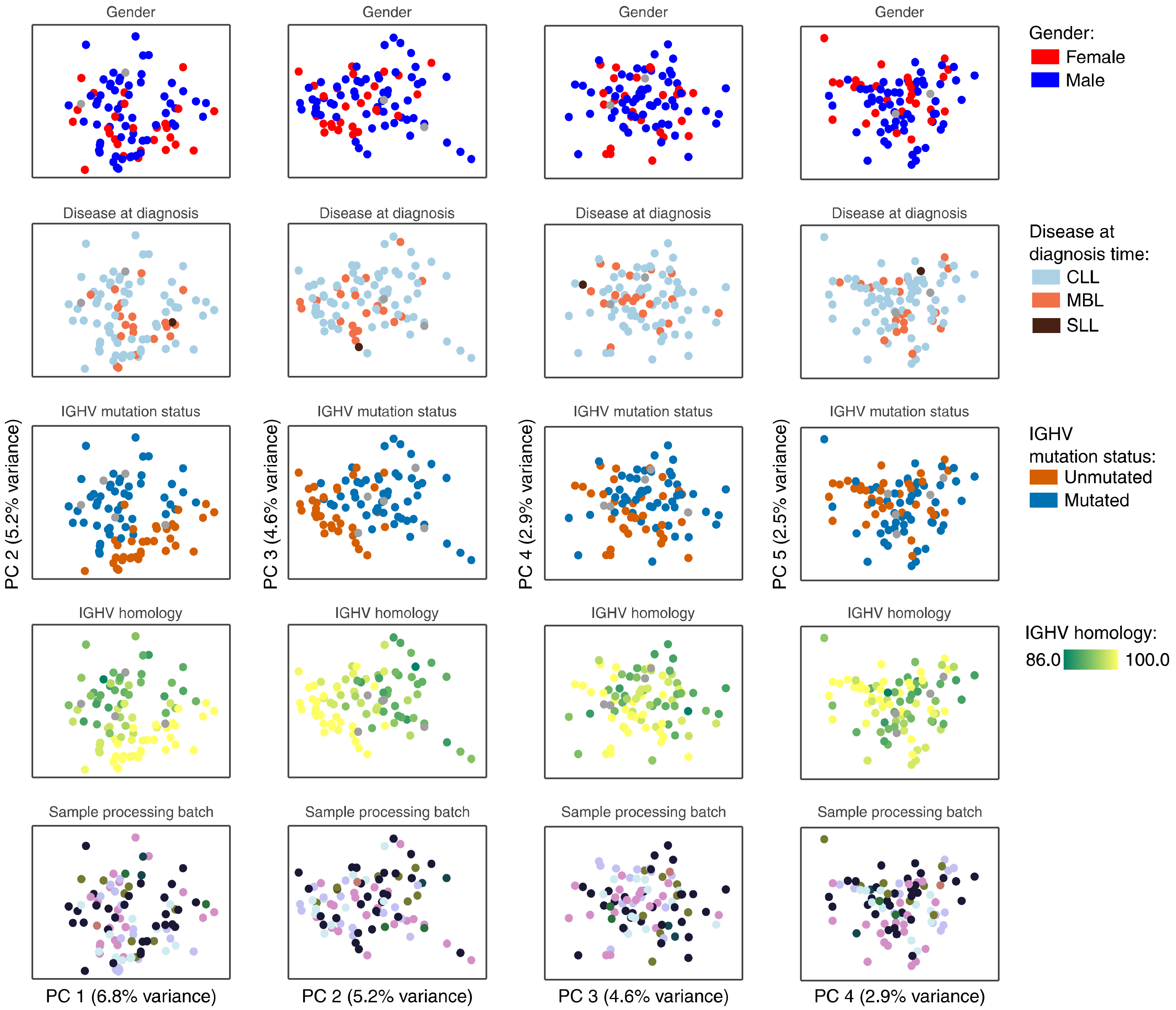

Supplementary Figure 6

Unsupervised analysis identifies IGHV mutation status as a key source of variation.

Principal component analysis based on the chromatin accessibility for all 88 samples at each of the 112,298 chromatin-accessible regions in the CLL cohort. The first five principal components are plotted, and samples are colored according to clinical annotations and molecular diagnostics data (top four rows) as well as the sample processing batch for the ATAC-seq experiments (bottom row).

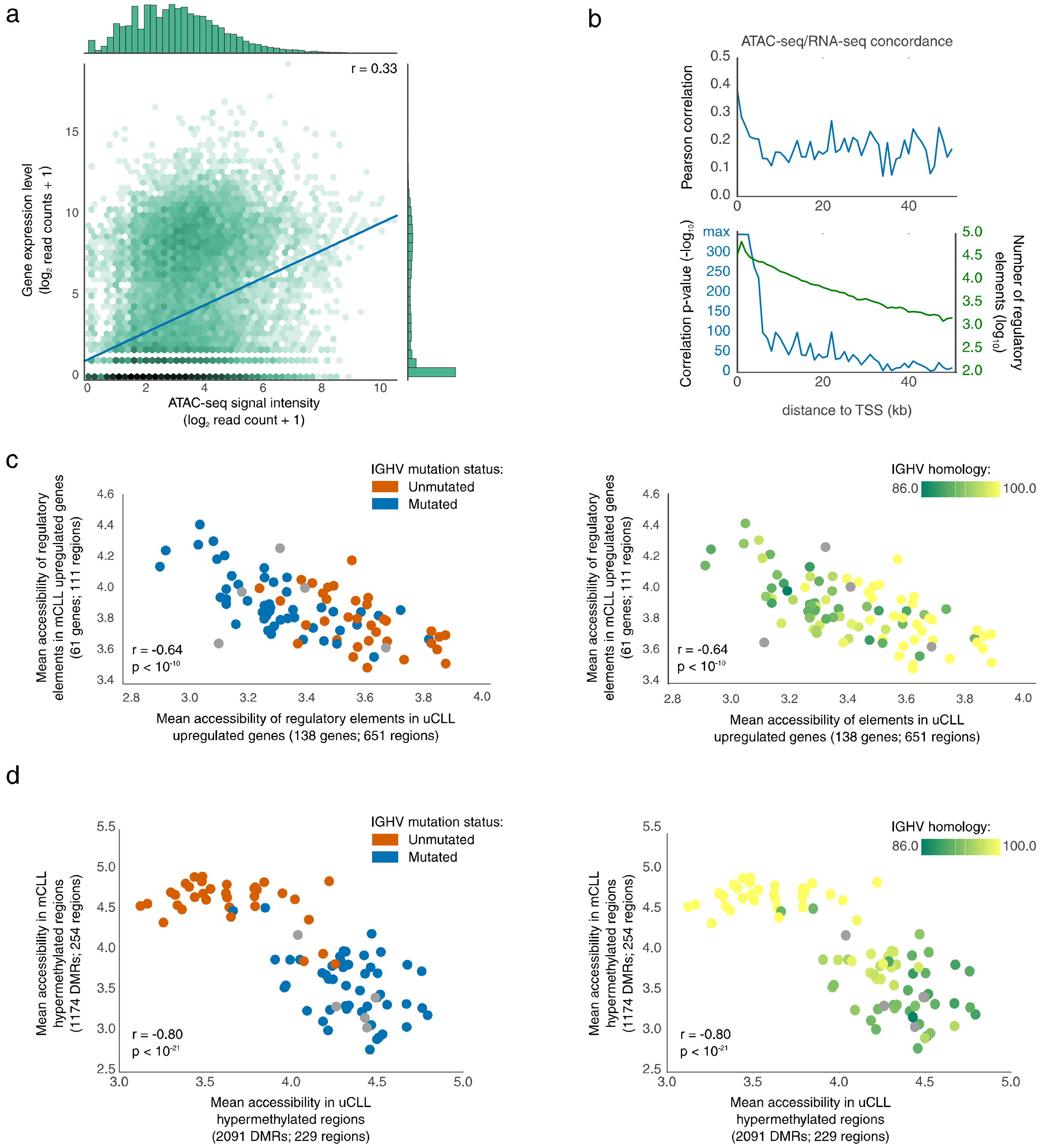

Supplementary Figure 7

Chromatin accessibility is linked to differences in gene expression and DNA methylation.

a) Hexbin scatterplot visualizing the weak correlation (Pearson's $r = 0.33$) between gene expression levels and the chromatin accessibility at associated regulatory regions. Shown are averages across ten samples with matched ATAC-seq and RNA-seq data. The color gradient is on a logarithmic scale. b) Pearson correlation (top) and significance of the association (bottom) between gene expression levels and chromatin accessibility values at associated regulatory regions, plotted over the distance of the accessible region to the gene's transcription start site. c) Mean chromatin accessibility across CLL-accessible regions associated with genes that were upregulated in *IGHV*-mutated or in *IGHV*-unmutated CLL. d) Mean chromatin accessibility in CLL-accessible regions that overlap with regions described as hypermethylated in *IGHV*-mutated or in *IGHV*-unmutated CLL (Kulis et al. 2012 Nature Genetics).

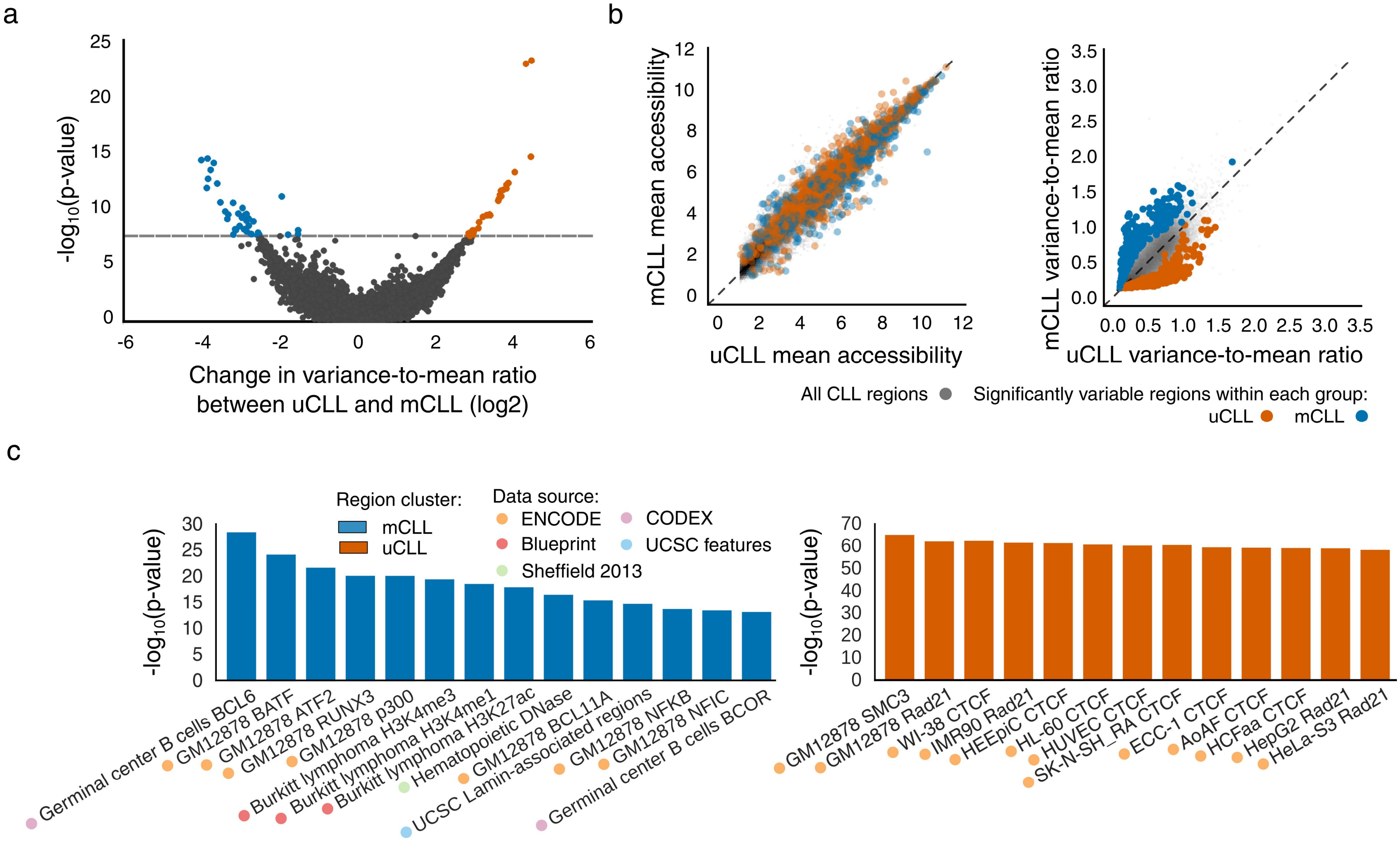

Supplementary Figure 8

Subtype-specific variable regions show characteristic enrichment patterns.

a) Differential variability between the mCLL and uCLL sample groups illustrated by each region's change in variance-to-mean ratio between groups (x-axis) and the *p*-value for variability within each group (y-axis). Blue and orange dots indicate significantly variable regions. b) Scatterplots of mean accessibility (left) and variance-to-mean ratio within each sample group (right). The plot on the left illustrates how significantly variable regions are dispersed across the accessibility range, rather than being strongly associated with differences in mean accessibility between the groups. The color coding is the same as in panel a. c) Most highly enriched region sets that significantly overlap with the differentially variable regions for mCLL (blue) and for uCLL (orange), based on LOLA analysis.

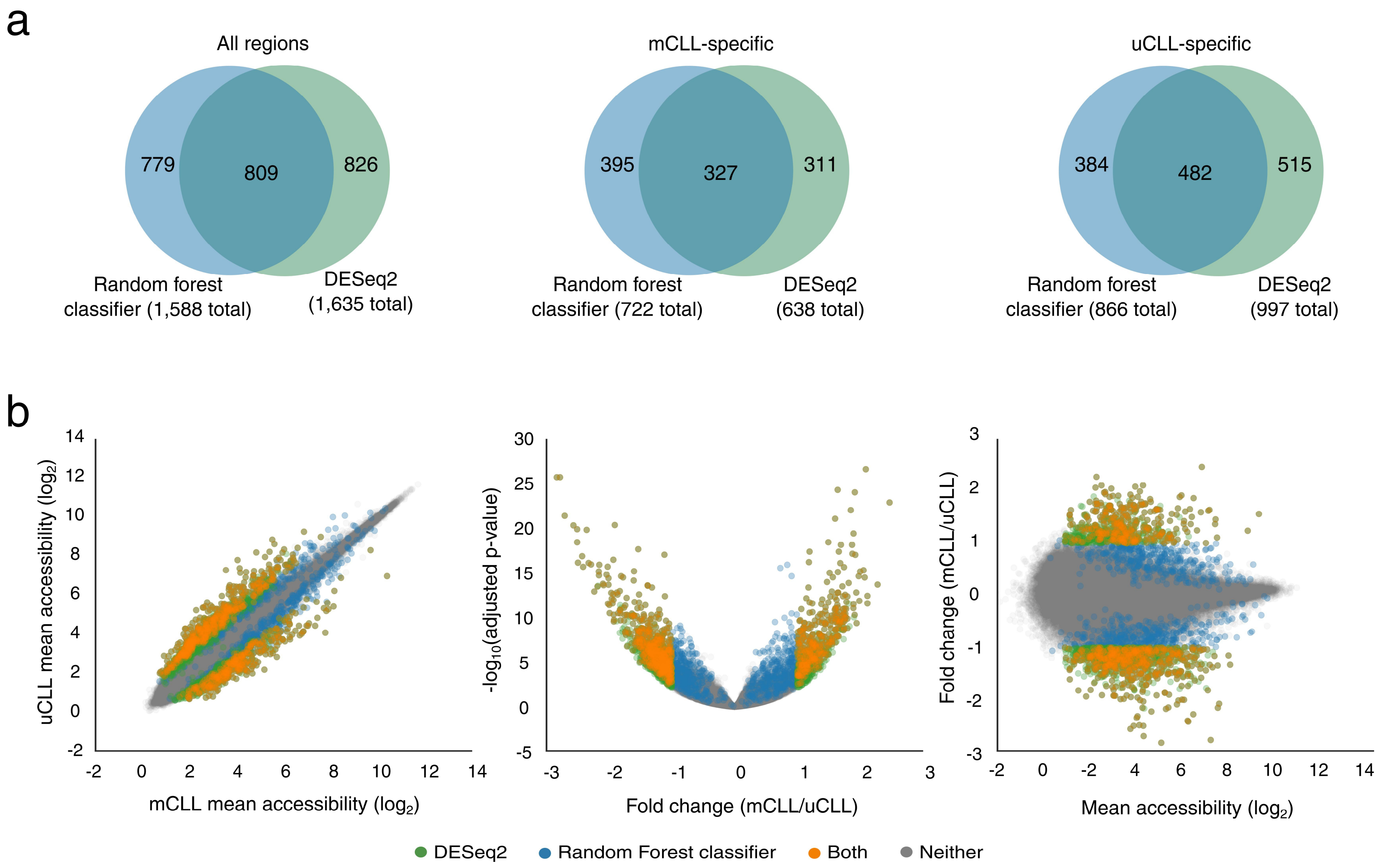

Supplementary Figure 9

Subtype-specific signature regions overlap with differential ATAC-seq peaks.

a) Venn diagrams showing the overlap between CLL subtype-specific regions identified by the machine learning analysis (left) compared to those identified by differential peak analysis between mCLL and uCLL using DESeq2. b) Scatterplot (left), volcano plot (center) and MA plot (right) comparing the two analytical approaches across all chromatin-accessible regions.

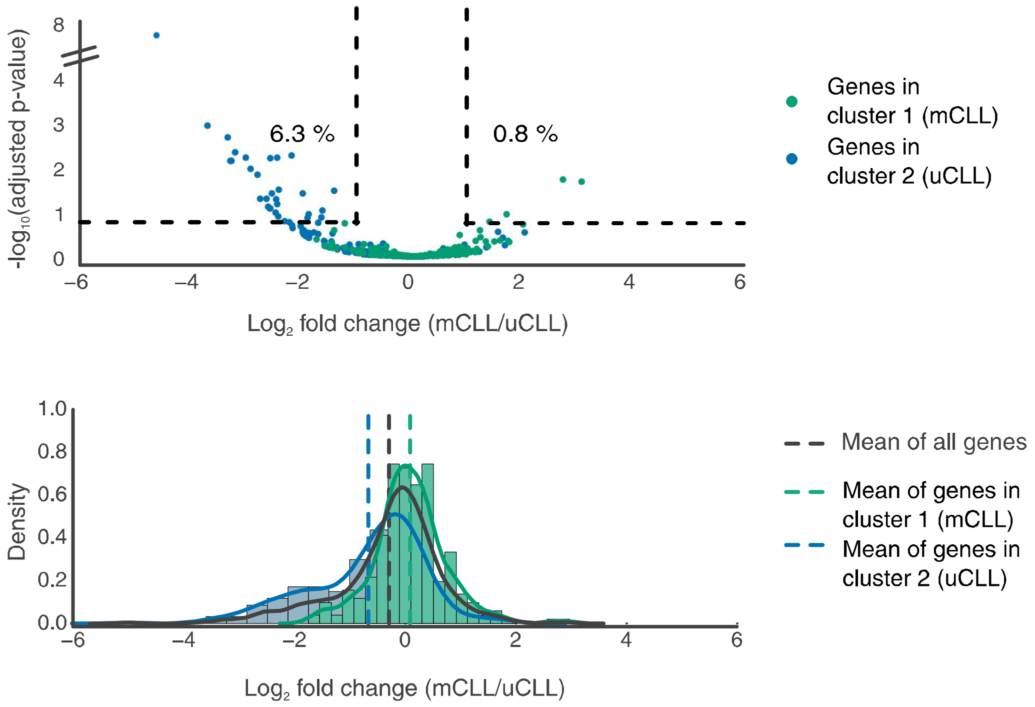

Supplementary Figure 10

Subtype-specific signature regions are weakly associated with differentially expressed genes.

Volcano plot (top) and histogram (bottom) showing gene expression differences between mCLL and uCLL samples for genes that are co-localized with subtype-specific signature regions. Percentage values are based on the number of genes that were significantly differentially expressed in the RNA-seq analysis.

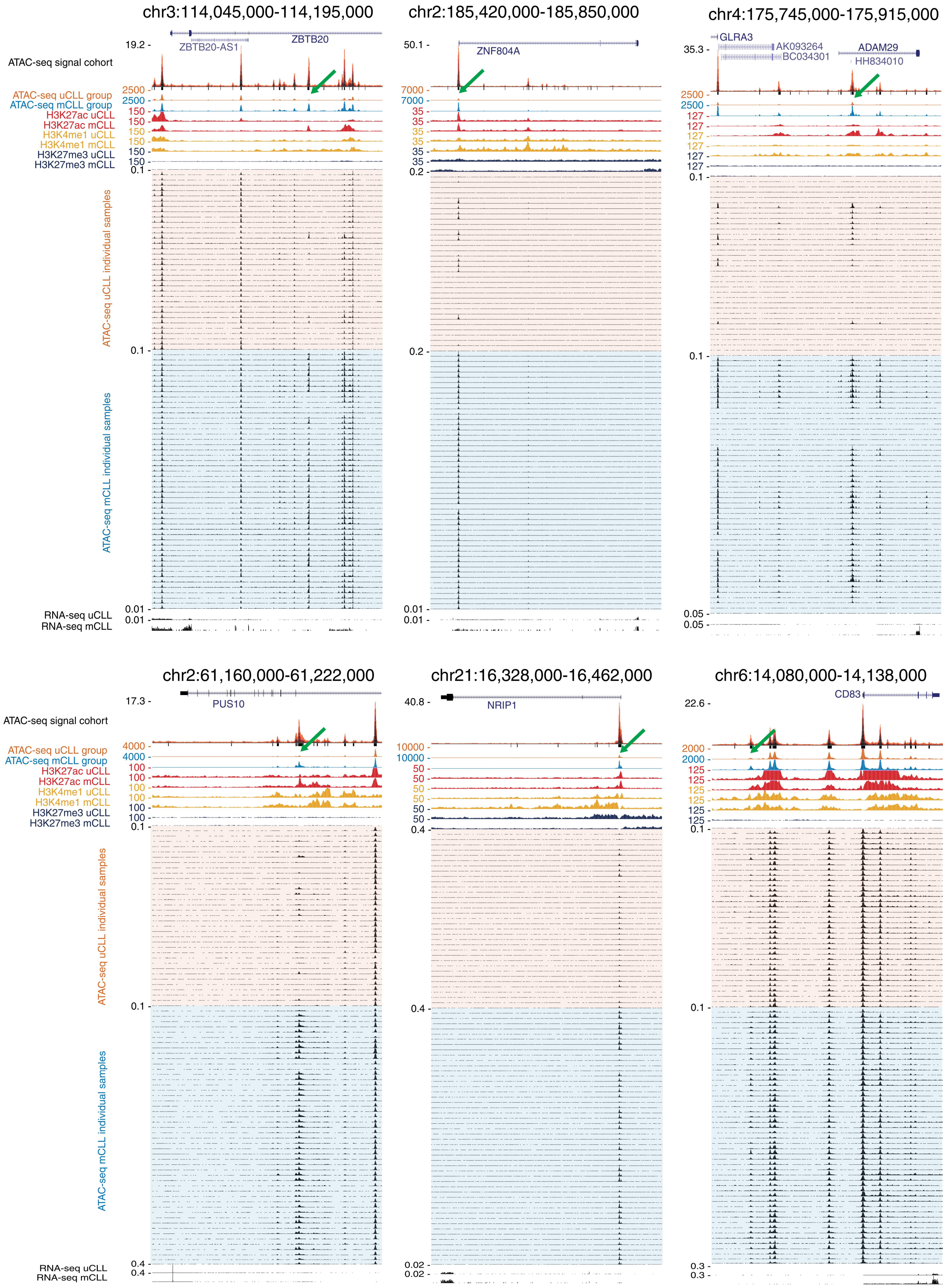

Supplementary Figure 11

Signature regions specific to mCLL show strong differences between disease subtypes but also heterogeneity within each subtype.

Genome browser plots for six gene loci that contain mCLL-specific signature regions (indicated by the green arrows). All ATAC-seq tracks were normalized by read depth to improve comparability between samples.

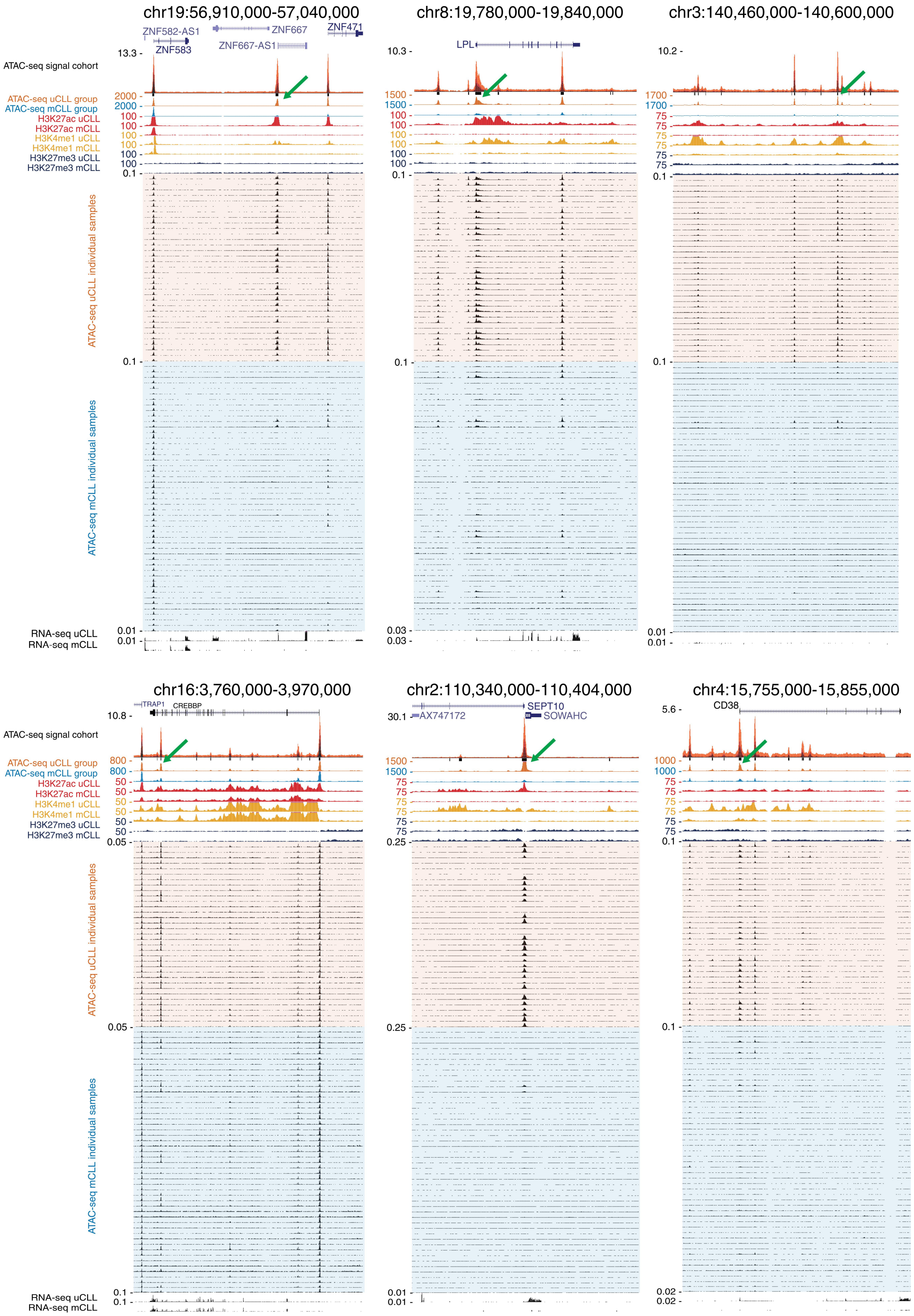

Supplementary Figure 12

Signature regions specific to uCLL show strong differences between disease subtypes but also heterogeneity within each subtype.

Genome browser plots for six gene loci that contain uCLL-specific signature regions (indicated by the green arrows). All ATAC-seq tracks were normalized by read depth to improve comparability between samples.

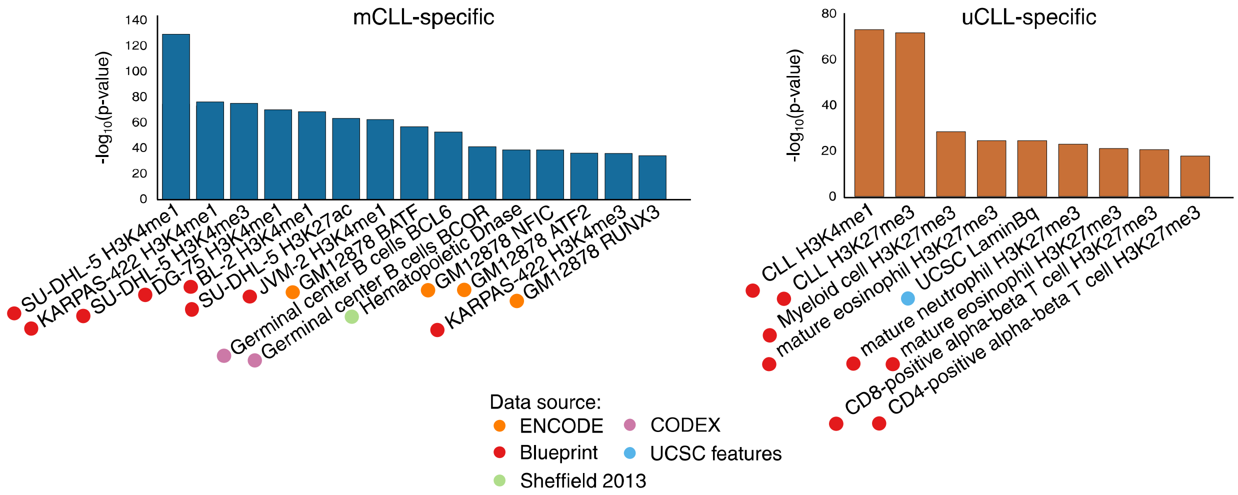

Supplementary Figure 13

Enrichment analysis for differential ATAC-seq peaks yields similar results as for the subtype-specific signature regions.

Complementing and validating the enrichment analysis shown in Figure 3g, this diagram lists the most highly enriched LOLA region sets for mCLL-specific (blue) and uCLL-specific (orange) differential peaks identified using DESeq2.

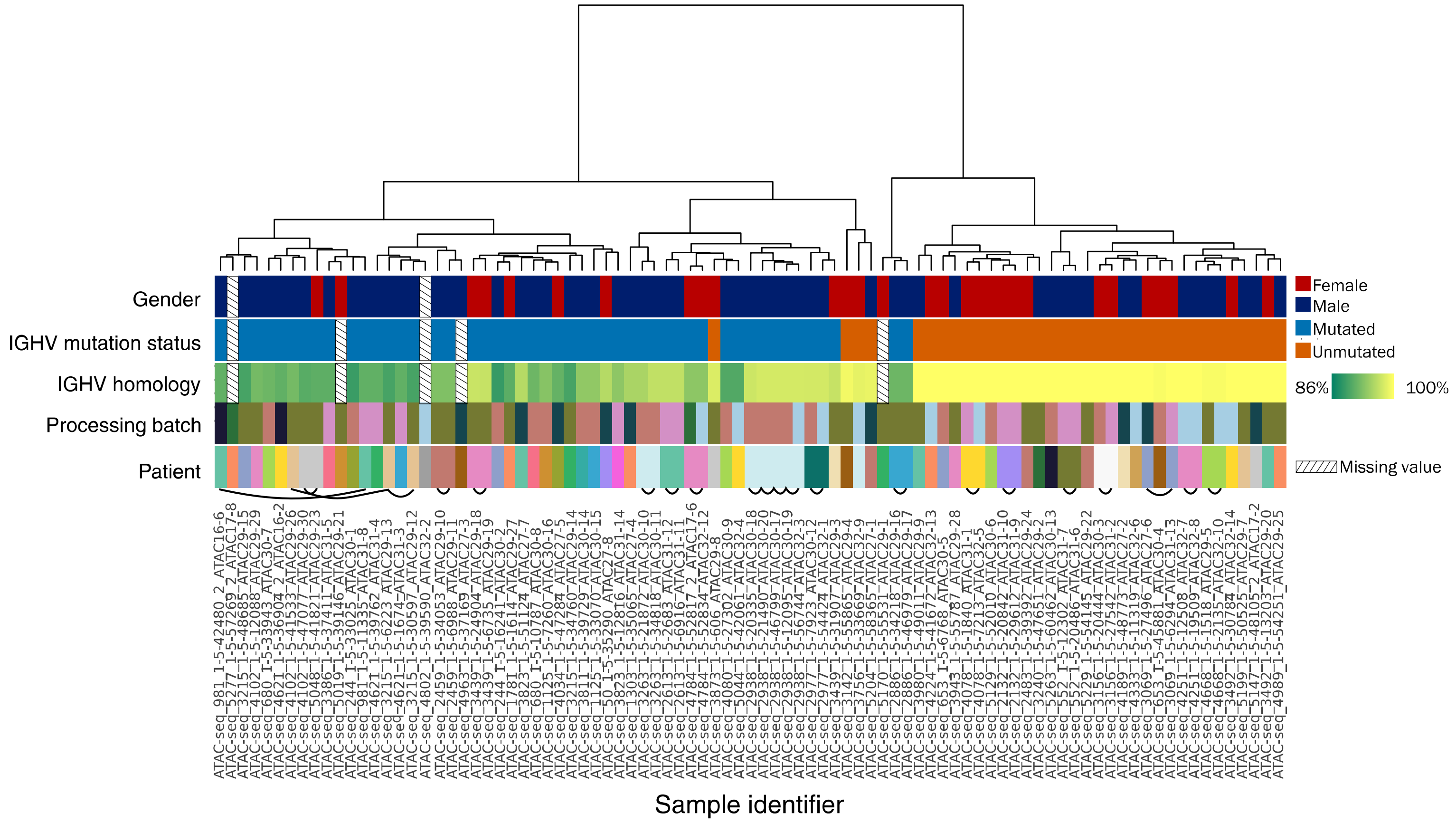

Supplementary Figure 14

Clustering based on CLL subtype-specific signature regions reflects IGHV mutation status.

Hierarchical clustering of all CLL samples based on sample-wise correlation of chromatin accessibility for the most discriminatory regions that were identified between the *IGHV*-mutated and the *IGHV*-unmutated disease subtype. The clustering tree is annotated with clinical data, and samples from the same patient are connected by curved black lines.

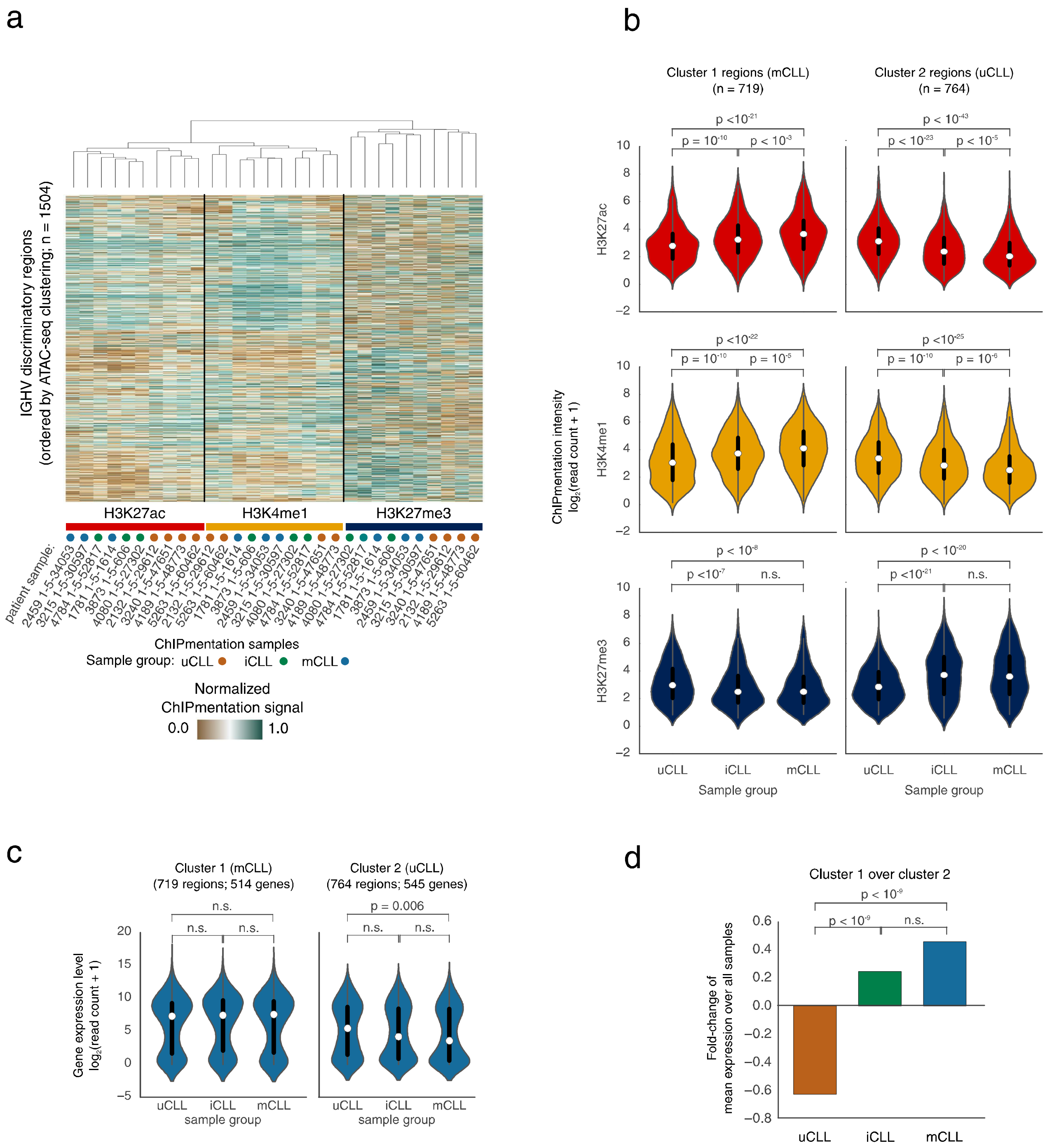

Supplementary Figure 15

Histone marks and gene expression confirm the intermediate character of the iCLL sample cluster.

a) Hierarchical clustering and heatmap visualizing the ChIPmentation signal for three histone marks (H3K4me1, H3K27ac, H3K27me3) in ten CLL samples comprising three disease subtypes (mCLL, iCLL, uCLL). Regulatory regions were selected and sorted in the same way as in Figure 3c. b) Violin plots showing the distribution of ChIPmentation levels for each histone mark in the same regulatory regions as in panel a, grouped by disease subtype. In all panels, significance was assessed using the Mann-Whitney *U* test, and comparisons with *p*-values above 0.05 were labeled as not significant (n.s.). c) Mean gene expression values for genes associated with the regulatory regions from panel a, grouped by disease subtype. d) Barplot showing the mean fold change of genes associated with regulatory elements in cluster 1 (mCLL regions) over genes associated with cluster 2 (uCLL regions) across all genes, grouped by disease subtypes. Significance was assessed using the Mann-Whitney *U* test, and comparisons with *p*-values above 0.05 were labeled as not significant (n.s.).

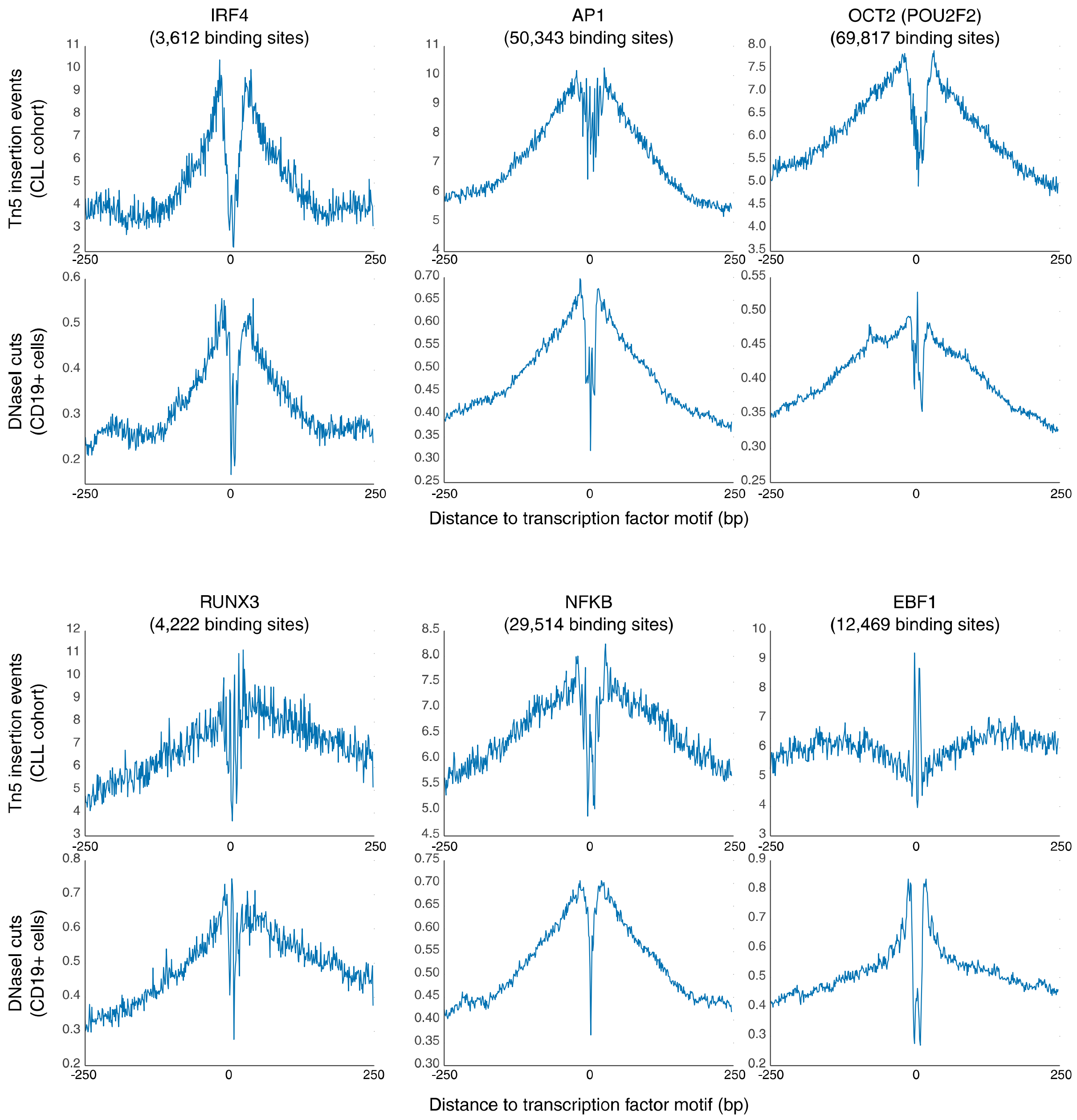

Supplementary Figure 16

Transcription factor footprints for ATAC-seq and DNase-seq are similar.

Footprinting diagrams showing the frequency of Tn5 transposase insertion events (for ATAC-seq) and DNase I cutting sites (for DNase-seq, based on data for CD19+ B cells from the Roadmap Epigenomics project) across a 500 basepair window around DNA binding motifs of transcription factors involved in B cell development.

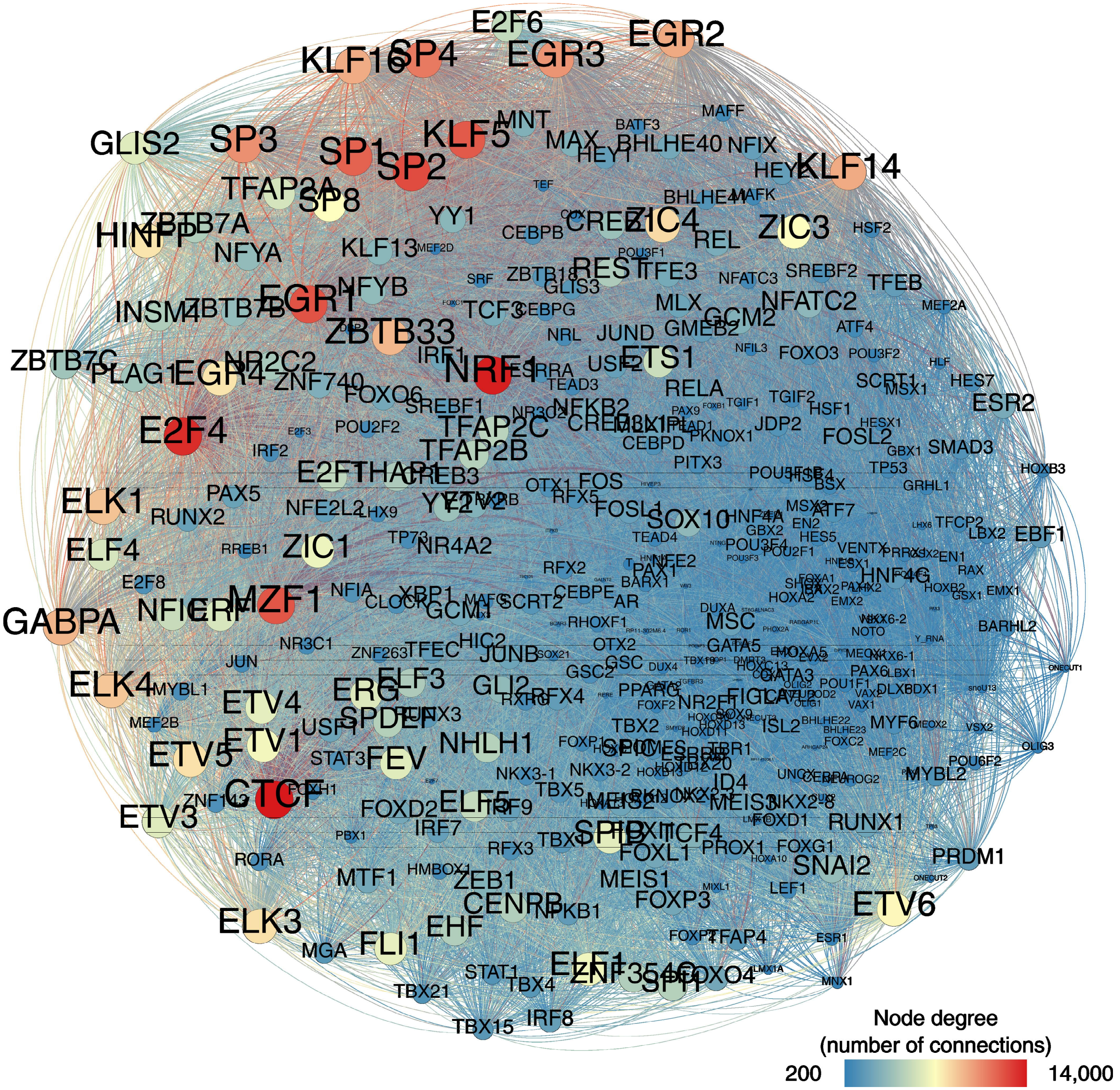

Supplementary Figure 17

Cohort-level gene regulatory network identifies transcription factors relevant to CLL.

Gene regulatory network of CLL inferred from footprint predictions of transcription factor binding, based on the ATAC-seq data of all CLL samples. Only nodes with more than 200 connections are shown.

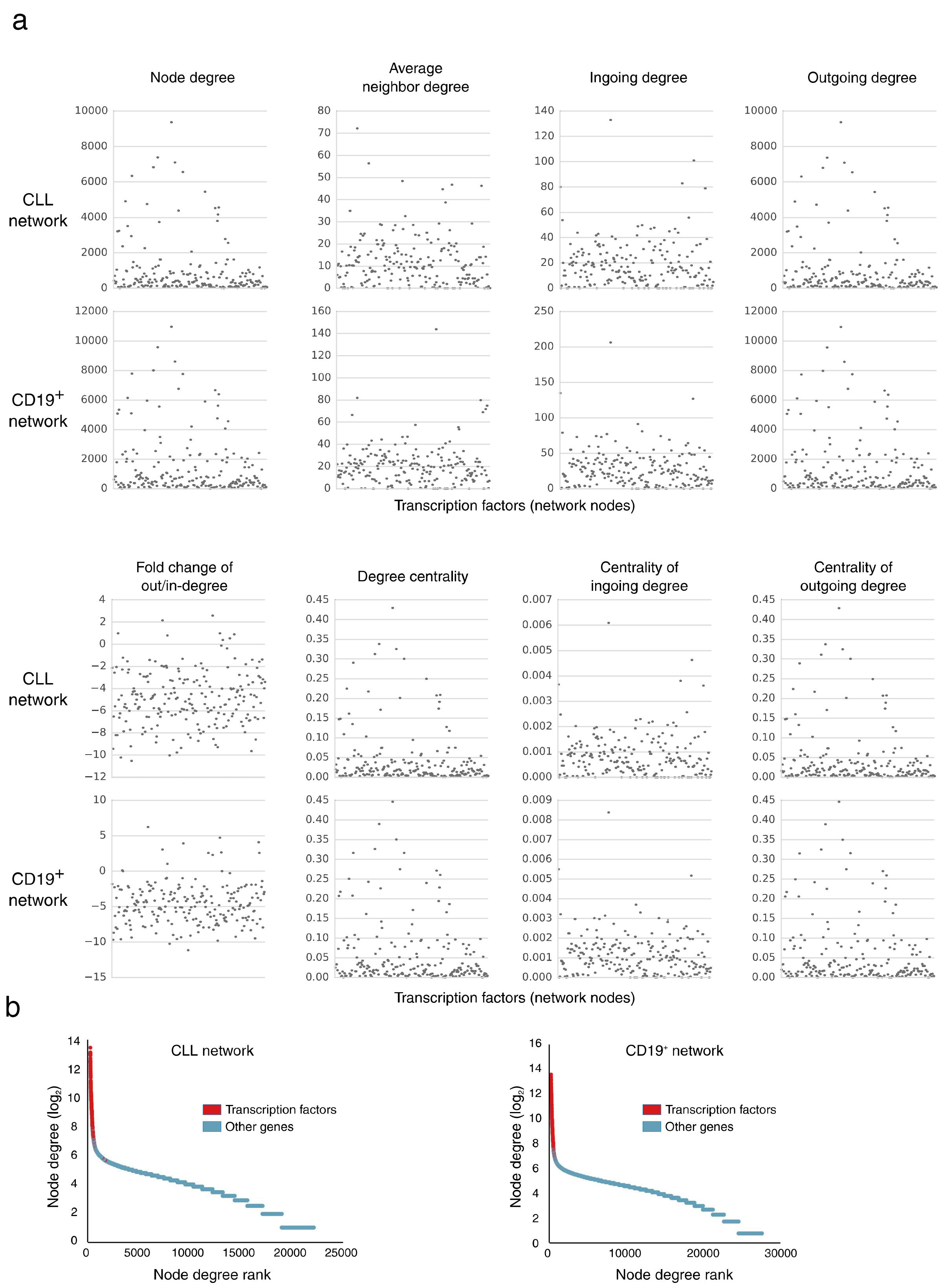

Supplementary Figure 18

Footprinting-based gene regulatory networks for ATAC-seq in CLL and DNase-seq in B cells show similar properties.

a) Structural properties of gene regulatory networks inferred from ATAC-seq data for the CLL cohort and from DNase-seq data for CD19+ B cells. b) Number of connections for all genes in the two gene regulatory networks (transcription factors are shown in red).

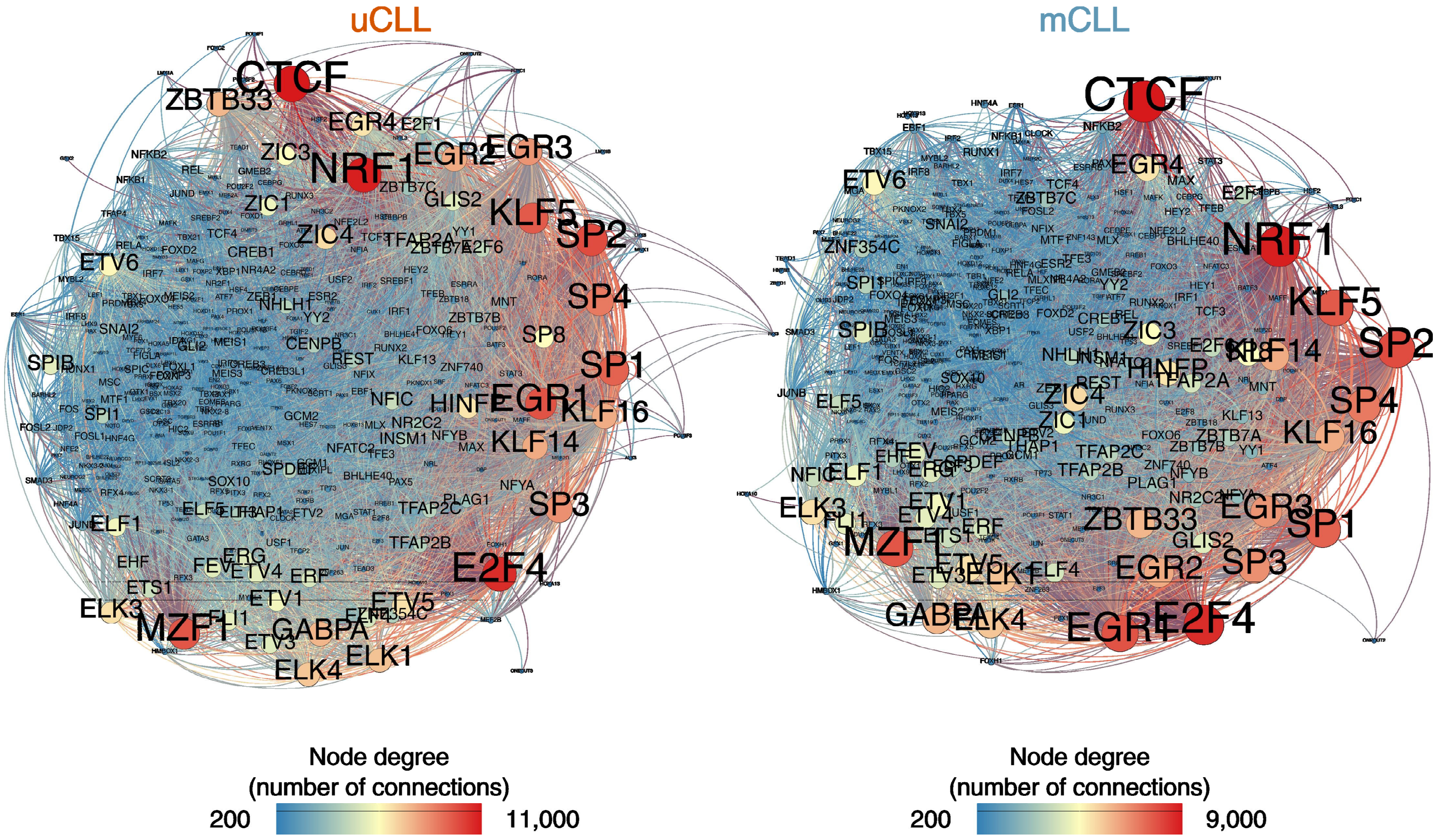

Supplementary Figure 19

Gene regulatory networks for mCLL and uCLL samples are globally similar.

Gene regulatory networks inferred based on the *IGHV*-unmutated samples (uCLL, left) and based on the *IGHV*-mutated samples (mCLL, right). Only nodes with more than 200 connections are shown.

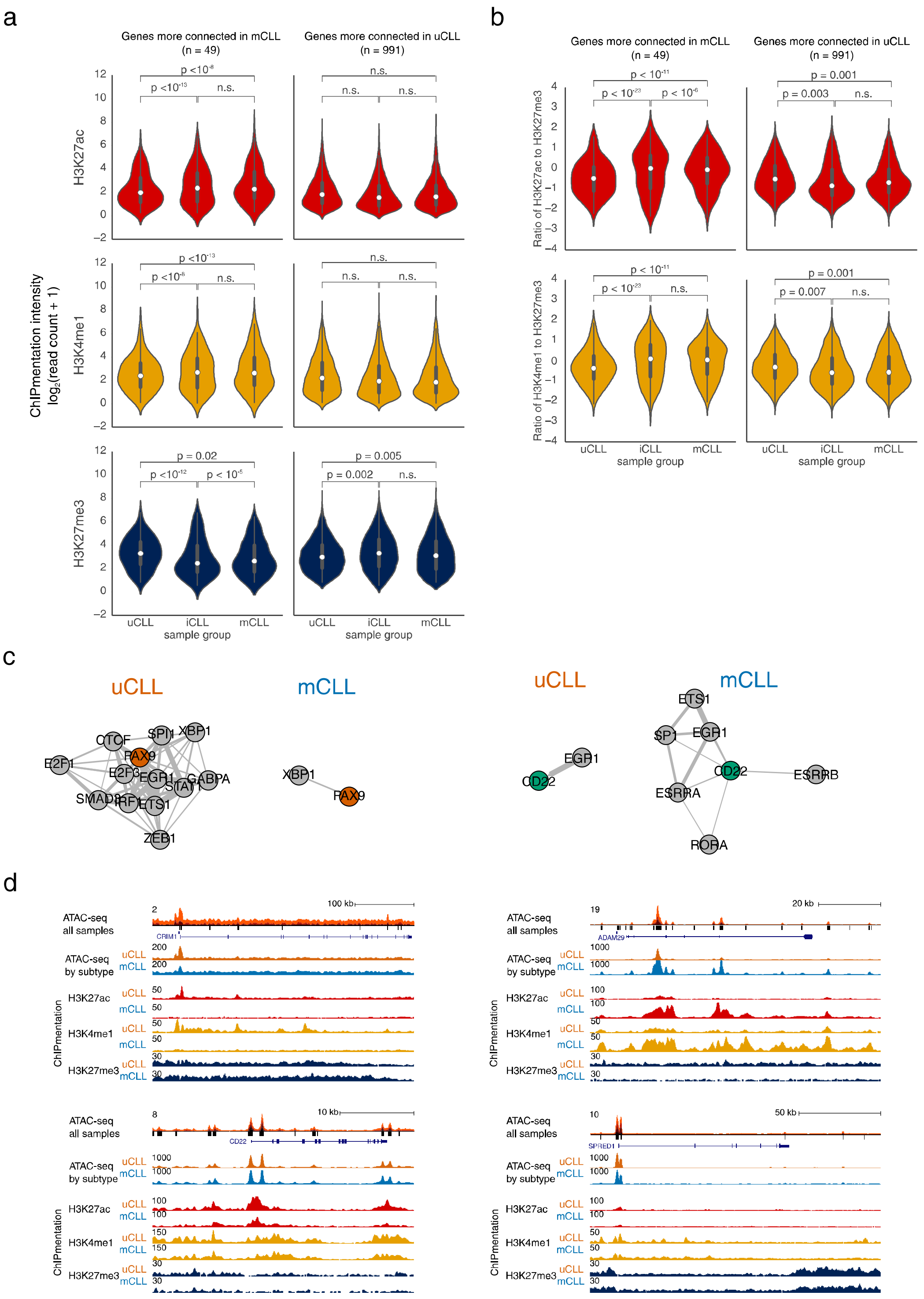

Supplementary Figure 20

Disease subtype-specific networks detect differentially regulated genes and genomic regions.

a) Violin plots showing the distribution of ChIPmentation levels for each histone mark in regulatory regions associated with genes that are differentially connected between the subtype-specific networks. b) Violin plots showing the ratio between the ChIPmentation signal for histone marks associated with active (H3K4me1, H3K27ac) over repressed (H3K27me3) chromatin. c) Subnetworks with the neighbors of PAX9 and CD22, shown separately for the mCLL and uCLL networks. Edge width indicates the strength of the connection as measured by the calculated interaction score. d) ATAC-seq and ChIPmentation signal for three histone marks at representative differentially connected genes between the mCLL and uCLL networks. In panel a and b, significance was assessed using the Mann-Whitney U test, and comparisons with p-values above 0.05 were labeled as not significant (n.s.).
